# Impact of Immunity on Coronary Artery Disease: An Updated Pathogenic Interplay and Potential Therapeutic Strategies

**DOI:** 10.3390/life13112128

**Published:** 2023-10-27

**Authors:** Nicola Laera, Paolo Malerba, Gaetano Vacanti, Simone Nardin, Matteo Pagnesi, Matteo Nardin

**Affiliations:** 1 Department of Clinical and Experimental Sciences, University of Brescia, 25123 Brescia, Italy; pmalerba92@gmail.com; 2 Second Medicine Division, Department of Medicine, ASST Spedali Civili di Brescia, 25123 Brescia, Italy; 3 Division of Medicine, Department of Medicine, ASST Spedali Civili di Montichiari, 25018 Montichiari, Italy; 4 Medical Clinic IV, Department of Cardiology, Municipal Hospital, 76133 Karlsruhe, Germany; gaetano.vacanti@klinikum-karlsruhe.de; 5 U.O. Clinica di Oncologia Medica, IRCCS Ospedale Policlinico San Martino, 16132 Genova, Italy; simone.nardin.94@gmail.com; 6 Department of Internal Medicine and Medical Sciences, School of Medicine, University of Genova, 16126 Genova, Italy; 7 Division of Cardiology, ASST Spedali Civili of Brescia, 25123 Brescia, Italy; m.pagnesi@gmail.com; 8 Department of Biomedical Sciences, Humanitas University, Via Rita Levi Montalcini 4, Pieve Emanuele, 20090 Milan, Italy; matteo.nardin.89@gmail.com; 9 Third Medicine Division, Department of Medicine, ASST Spedali Civili di Brescia, 25123 Brescia, Italy

**Keywords:** coronary artery disease, atherosclerosis, innate immunity, trained immunity, checkpoints inhibitors

## Abstract

Coronary artery disease (CAD) is the leading cause of death worldwide. It is a result of the buildup of atherosclerosis within the coronary arteries. The role of the immune system in CAD is complex and multifaceted. The immune system responds to damage or injury to the arterial walls by initiating an inflammatory response. However, this inflammatory response can become chronic and lead to plaque formation. Neutrophiles, macrophages, B lymphocytes, T lymphocytes, and NKT cells play a key role in immunity response, both with proatherogenic and antiatherogenic signaling pathways. Recent findings provide new roles and activities referring to endothelial cells and vascular smooth muscle cells, which help to clarify the intricate signaling crosstalk between the involved actors. Research is ongoing to explore immunomodulatory therapies that target the immune system to reduce inflammation and its contribution to atherosclerosis. This review aims to summarize the pathogenic interplay between immunity and CAD and the potential therapeutic strategies, and explore immunomodulatory therapies that target the immune system to reduce inflammation and its contribution to atherosclerosis.

## 1. Introduction

**Figure 1:**
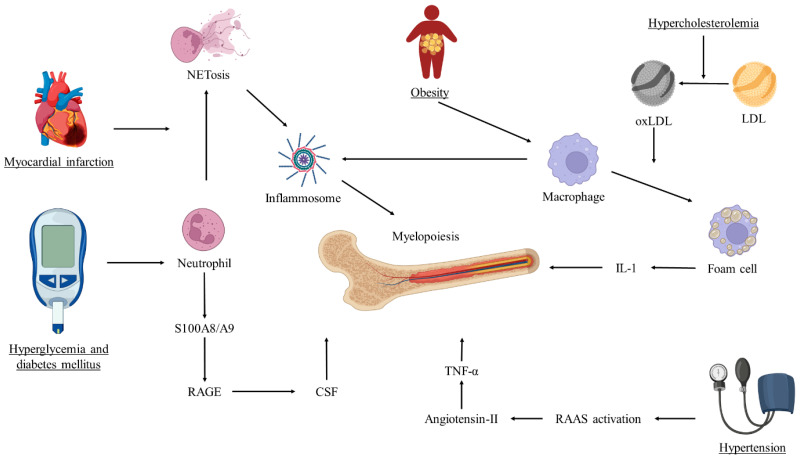
Crosstalk between cardiovascular risk factors and myelopoiesis. The figure depicts how the traditional cardiovascular risk factors impact myelopoiesis, further sustaining the inflammation related to the progression of atherosclerosis. CSF = colony-stimulating factor; IL-1 = interleukin-1; LDL = low-density lipoprotein; NET = neutrophil extracellular trap; oxLDL = oxidized low-density lipoprotein; RAAS = renin-angiotensin-aldosterone system; RAGE = receptor of advanced glycation end-products; TNF-α = tumor necrosis factor-α.

Atherosclerosis is the leading cause of mortality worldwide and one of its main manifestations is ischemic heart disease, mostly determined by the involvement of the coronary arteries, known as coronary artery disease (CAD). CAD is primarily a result of the buildup of atherosclerosis within the coronary arteries, progressively leading to the narrowing of the vessel lumen, thus reducing the blood flow with consequent ischemia. Upon stimulation by deposited lipids and damaged endothelium, innate and adaptive immune cells are activated and recruited to initiate plaque development [1]. The role of the immune system in CAD has been shown during the last few years to be complex and multifaceted. Immunity plays a key role in several types of pathways. First, inflammation is a key part of atherosclerosis [2]. Both innate and adaptive immune responses are activated to remove dead and apoptotic cells, facilitate scar formation, and promote angiogenesis [3]. Innate immunity includes neutrophils and macrophages, which can directly phagocytose dead cells and debris. They also play a role in releasing cytokines and other molecules that promote an inflammatory response [4,5]. Adaptive immunity involves T cells and B cells, which can recognize and respond to specific antigens. This response can also shape the overall immune response to an injury [6,7]. However, in the context of atherosclerosis, this inflammatory response can become chronic and lead to plaque formation. Immune cells, particularly macrophages, play a pivotal role in the uptake of low-density lipoprotein (LDL), mostly oxidized-LDL (ox-LDL), leading to the formation of foam cells within the atherosclerotic plaque [5]. The increasing amount of evidence deserves further critical considerations to be better outlined; that is, the aim of this review is a focus on the most recent advances related to immunity and atherosclerosis [8] (Figure 1).

## 2. Immune Cells Involved in CAD

### 2.1. Neutrophils

Neutrophils are the most abundant leucocytes and play a significant role in mediating sterile inflammation and injury through a variety of mechanisms [4,9]. Previous experimental works have elucidated their roles in atherosclerotic diseases like CAD and its ensuing complications, i.e., acute coronary syndrome and heart failure [9]. Early aortic lesions and rupture- or erosion-prone atherosclerotic plaques show a significant presence of neutrophils [10]. A high peripheral neutrophil count directly relates to the degree of atherosclerosis in coronary arteries [11], infarct size, and declines in left ventricular ejection fraction [12,13]; the neutrophil-to-lymphocytes ratio raises clinical attention, due to its potential relationship with CAD [14]. Recent evidence also suggests a role for neutrophils in the activation of reparative processes [15]. In animal studies, for example, long-term depletion of neutrophils after myocardial infarction (MI) resulted in worsened cardiac function and increased fibrosis [15]. Neutrophils are paramount for innate immune response as they are the first responders in our defense against invading pathogenic microorganisms [4]. However, in sterile inflammatory conditions, activation of neutrophils may have detrimental effects on host tissues and therefore their homeostasis must be tightly regulated [16]. Interestingly, most clinically recognized cardiovascular risk factors contribute to enhanced granulopoiesis, e.g., the production of neutrophils in the bone marrow [4]. Once production bursts are triggered by those risk factors, neutrophils play a leading role in the initiation and evolution of unstable atherosclerotic plaques. At sites of disturbed blood flow and increased shear stress, they dysregulate vascular endothelial cells (ECs) and trigger leucocyte arrest [17], setting the stage for atherosclerosis [18]. Further release of granule proteins degrades the extracellular matrix (ECM), leading to extra-adhesion of monocytes, vascular hyperpermeability, and transfer of LDL particles [19]. Through the nucleotide-binding oligomerisation domain-like receptor pyrin domain-containing protein 3 (NLRP3) inflammasome signaling, activated neutrophils in the atheroma undergo neutrophil extracellular trap (NET) formation (NETosis), a type of cell death [20] using a network of ECM containing a variety of granule proteins. Their release decreases the stability of atherosclerotic plaques and contributes to thrombus formation through a variety of mechanisms [21,22,23,24]. Not only do neutrophils play an active role in the genesis of atherosclerotic plaques, they also mediate their consequences to the target organs. Rupture of an atherosclerotic plaque can lead to obstruction of blood circulation resulting in ischaemic death of tissues that immediately triggers an acute inflammatory response, led mostly by neutrophils [21]. Attracted by cellular debris released by dead cells, neutrophils massively infiltrate the infarct area within hours [22]. At the site of injury, activated neutrophils generate and release reactive oxygen species, proteases, and NETs [23] which promote cardiomyocyte apoptosis, degrade ECM [24], lead to leucocyte infiltration, and prime the NLRP3 inflammasome, which then stimulates granulopoiesis in the bone marrow, leading to a vicious circle of the neutrophil infiltration circle and maladaptive remodeling [23]. Neutrophils are also involved in the modulation of the healing and remodeling response: the protein S100A8/A9 in NETs activated macrophages to phagocyte dead cells [25]. The transcriptional profile of neutrophils changes from a pro-inflammatory profile to an anti-inflammatory profile, initiating the reparative process mostly by dedifferentiating cardiomyocytes and promoting the accumulation of reparative macrophages [26,27]. Finally, a subset of pro-angiogenesis neutrophils able to control blood vessel growth, a known mechanism of tissue regeneration after injury, has been recently identified [28,29].

This dichotomous and apparently paradoxical effect of neutrophil activity in atherosclerotic diseases highlights the difficulty of researching beneficial therapeutical strategies involving neutrophils and calls for an expertly tailored approach to the matter. In the short term, stunning inhibition of neutrophils such as through beta-adrenergic antagonists as metoprolol or through inhibition of S100A8/A9 has been shown to reduce infarct size and increase left ventricular ejection fraction [30,31]. However, long-lasting depletion of neutrophils or even long-term inhibition of S100A8/A9 resulted in worse cardiac function and increased fibrosis [25,32]. Identifying the right window to effectively suppress the inflammatory functions of neutrophils while retaining their reparative functions could pave the way for important therapeutical applications.

### 2.2. Macrophages

Just as for neutrophils, the role of macrophages in inflammation, particularly in its cardiovascular aspects, is multifaceted [5,33]. Many studies have shown their ability to trigger and drive robust and damaging inflammatory responses [34], while others have shown their involvement in tissue repair and even cardiac regeneration [5,35,36]. This seems to be because different macrophage populations mediate different responses [37]. Furthermore, due to their high plasticity, they can adopt different phenotypes in response to varying stimuli and environments, a process called polarization [33]. Classically, cardiac macrophages have been categorized according to inflammatory states and cell surface markers into pro-inflammatory (M1) and anti-inflammatory (M2) subsets [26]. 

Newly discovered subsets of macrophages with mixed M1/M2 cell surface markers have challenged the adequacy of the classification [38]. In vitro, culture of human macrophages revealed considerable deviation from the M1/M2 spectrum when in contact with a cardiovascular relevant stimulus, e.g., free fatty acids or high-density lipoprotein (HDL) [39]. Furthermore, in vivo studies of murine atherosclerosis showed how, for example, inflammatory macrophages express cell surface markers like CD206, typically used to define the M2 anti-inflammatory subset [40]. In other words, the traditional classification of macrophages into M1 and M2 phenotypes does not fully capture the diversity of the population in vivo.

An alternative approach would classify macrophages according to developmental lineage, transcriptional factors, and recruiting dynamic as well as cell surface markers [5]. Through this classification, new functionally distinct cardiac macrophage populations have been elucidated. The adult heart contains three distinct populations of macrophages to the expression of C-C chemokine receptor type 2 (CCR2) and major histocompatibility complex class II (MHC-II): CCR2^-^MHC-II^low^, CCR2^-^MHC-II^high^, and CCR2^+^ MHC-II^high^. 

CCR2^-^MHC-II^low^ and CCR2^-^MHC-II^high,^ are long-lived, derive from embryonic progenitors maintained through the proliferation of local macrophages in the heart, and represent the vast majority at steady state [5]. They show an enhanced capacity to phagocyte death cardiomyocytes and exhibit a low inflammatory profile [41,42], while CCR2^-^MHC-II^high^ can, in vitro, elicit an important inflammation response, for example through antigen-presenting cells (APC) to T-cells [42,43]. CCR2^+^ MHC-II^high^ are relatively short-lived and derive exclusively from circulating monocytes [5]. At a steady state, their function is still unclear. Following an MI, however, the presence of mitochondrial deoxyribonucleic acid (DNA) and alarmins from dying cardiomyocytes activates a vast array of proinflammatory genes in this subpopulation of macrophages, for example in the NLRP3 pathway, involved in neutrophil-associated inflammatory response, as previously stated [44]. In the first acute response to ischemic injury (first 4–7 days) CCR2^-^MHC-II^low^ and CCR2^-^MHC-II^high^ continue to show their classical phagocytic, non-inflammatory function in the lesion area [26,45]. This triggers apoptosis of all resident macrophages and by 24 h post-MI they are almost completely absent [34]. At the same time, abundant blood monocytes infiltrate the lesion area and differentiate into pro-inflammatory CCR2^+^ MHC-II^high^ [34]. The fact that inhibition of monocyte extravasation into the cardiac tissue decreases macrophage numbers and improves cardiac physiology, highlights the importance of this population of macrophages in the adverse post-MI response [46]. In the reparative phase (days 5–14), monocytes differentiate into CCR2^-^MHC-II^high^ macrophages as opposed to CCR2^+^ MHC-II^high^ [47]. This switch in polarisation seems to be determined by changes in the local ischemic region: the infarct microenvironment is initially filled with early pro-M1 mediators, like interferon-γ (IFN-γ) and the granulocyte-macrophage colony-stimulating factor (GM-CSF), which trigger the initial differentiation into CCR2^+^ MHC-II^high^ macrophages and later with pro-M2 factors, like interleukin (IL) 10 and transform growth factor- β (TGF-β), stimulating differentiation into CCR2^-^MHC-II^high^ macrophages [48,49]. These promote angiogenesis and scar formation and regulate the ECM microenvironment [47,50,51], orchestrating the fine mechanisms leading to tissue modelling and healing. 

The mechanistic aspects of this flexible macrophage polarization are still poorly understood [52]. In the past 5 years, several studies have suggested an extensive epigenetic and transcriptional crosstalk between pro-inflammatory and anti-inflammatory signaling [53,54]. Responding to local stimuli, macrophages not only react at a transcriptional level [55], mounting the real-time response but they also adopt unique and permissive epigenetic changes, creating a cellular memory [56]. This memory enables the cells to launch a faster response upon reactivation, changing the macrophage activation state [52]. This allows a potentially more efficient response to pathological stimuli [57,58] but makes the system also prone to the dysregulation responsible for the clinical disease [52,59]. The tight control by transcription factors and epigenetic modifiers makes these pathways in macrophages a promising therapeutic target for inflammation-driven diseases.

### 2.3. Natural Killer T Lymphocytes: New Actors for an Old Disease?

Natural Killer T (NKT) cells have been the subject of increasing research about their role in the immune response to atherosclerosis and CAD [60]. NKT cells can be broadly categorized into two main subsets: Type I (invariant) and Type II NKT cells; type I NKT cells are the most well-studied subset and are characterized by their invariant T-cell receptor (TCR) alpha chain combined with a limited set of beta chains. These cells recognize glycolipid antigens such as α-galactosylceramide; they can rapidly produce a wide spectrum of cytokines, making them versatile regulators of immune responses. Type II NKT cells are less well-defined than Type I NKT cells and exhibit more distinct TCRs. They recognize a broader range of lipid antigens, including sulfatides, phospholipids, and glycolipids. Their functions are less clear, but they may also influence immune responses in various contexts [61,62]. The activation of these lipid-reactive NKT cells involves the interaction between lipid antigens, both endogenous and exogenous, and the nonclassical major histocompatibility complex class I (MHC-I) molecules of the cluster of differentiation (CD) 1 family on APC [63]. These lipid antigens bind to specific TCRs on T-cell subsets, including NKT cells. The structures of molecules in the CD1 family have been studied to understand how these lipid antigens associate with them. Group I CD1 molecules present lipid antigens from microbes and self-lipids to T cells, while group II CD1 molecules, specifically CD1d, present lipids to NKT cells. These CD1-presenting molecules are found on APC-like dendritic cells (DC), macrophages, and B cells, all of which play a role in the development of atherosclerotic lesions [64,65].

The activation of NKT cells can occur through various pathways. When an antigen is presented by CD1d molecules, a subset of NKT cells called invariant NKT cells (iNKT) respond rapidly by releasing cytokines, like helper T cells [66,67]. These cytokines have the potential to influence the development of atherosclerotic lesions in multiple ways [68,69]. The cytokines secreted by activated iNKT cells within the lesion may affect the response of other cells involved in the immune system, both innate and adaptive. In addition to antigen presentation by CD1d molecules, NKT cells can also be activated through a CD1d-independent pathway: DC or macrophages can be activated by Toll-like receptor (TLR) ligands, which then produce cytokines like IL-12, IL-18, or type I interferons. These cytokines can activate NKT cells without the involvement of CD1d molecules [70]. Moreover, TLR2 and TLR4 activation have been linked with atherosclerosis [71,72]. The effects of iNKT cells have been observed in studies conducted on mice. These studies administer different diets to mice to examine the impact of varying levels of iNKT cell activation or quantity. For instance, in mouse models treated with a Western-type diet, increased iNKT cell activity leads to increased plaque formation on the aortic root [73]. Conversely, in mice with genetically iNKT-deficient cells, an opposite effect on atherosclerotic lesions was shown [74].

NKT cells therefore play a crucial role in the immune response to CAD and atherosclerosis. The activation of these cells can occur through various pathways, including antigen presentation by CD1d molecules and CD1d-independent pathways. The cytokines released by activated NKT cells can influence the development of atherosclerotic lesions and modulate the response of other immune cells. Further research is required to fully understand the complex mechanisms by which NKT cells contribute to these diseases.

### 2.4. B Lymphocytes

B cells play a multifaceted role in atherosclerosis, depending on cellular differentiation: this process leads to subtypes B1 and B2 cell formation [75,76,77]. When naive B cells are exposed to a complex set of stimuli, they undergo differentiation and become antibody-secreting cells, specifically plasma blasts and plasma cells. In dyslipidemia, activated endothelium lining atherosclerotic plaques allow different immunoglobulins to enter the plaque area [78]. B1 cells are associated with producing antibodies, including IgM antibodies, with anti-inflammatory properties [79]. B1 cells may have a protective role in atherosclerosis by reducing inflammation and promoting plaque stability through their antibody production [80,81,82]. Contrary, B2 cells were initially viewed to be proatherogenic after preferential B2-cell depletion using CD20-targeted antibodies [83,84]. However, recent studies provide marginal zone B cells can conduct protective effects potentially secreting IgM [85].

In atherosclerosis, B cells produce antibodies directed against specific antigens present within the plaques. The most well-studied antibody target is ox-LDL. When LDL cholesterol particles become oxidized, they produce Oxidation-Specific Epitopes (OSEs) that can be recognized by the immune system. Anti-ox-LDL antibodies can promote inflammation and contribute to plaque formation by facilitating the uptake of ox-LDL by macrophages, leading to the formation of foam cells [86,87,88]. Advanced stages of plaque formation give rise to artery tertiary lymphoid organs, such as those found in the adventitia, where plasma cells are formed within the plaque. This leads to the production of immunoglobulins in the adventitia. To support this, atherosclerotic plaques contain immunoglobulins specific to different OSEs [89]. A substantial number of IgM antibodies in our immune system can identify OSEs [90]. These epitopes can be found on ox-LDL, apoptotic cells, and microvesicles. They also inhibit the pro-inflammatory responses of macrophages triggered by microvesicles [91]. Additionally, when macrophages are triggered by microvesicles, the IgM antibodies also play a role in reducing the pro-inflammatory responses of the macrophages. 

In contrast, IgG antibodies form immune complexes with ox-LDL, promoting inflammatory responses by macrophages [92]. IgE antibodies are known to have proatherogenic properties, as they stimulate macrophages and mast cells in both the plaque and perivascular area. Hamze et al. found that atherosclerotic plaques are rich in IgA and IgG, secreted by B cells during the inflammation process [93]. The involvement of IgA antibodies in atherosclerosis is still not well understood: a positive association between IgA and cardiovascular (CV) outcomes is reported, but functional roles have yet to be investigated [94].

B cells also produce various cytokines, including proatherogenic tumor necrosis factor -α (TNF-α) and antiatherogenic interleukin-10. Several studies on mouse models have described the protective role of B cells, remodeling the atheromatic plaque and increasing the lesion in case of B cell depletion [95,96]. However, there are different functional subsets of B cells, recognizing the heterogeneous population: both the proatherogenic and the antiatherogenic activities of various subsets are described [97,98]. While traditionally thought of as primarily involved in the production of antibodies, B cells also have antibody-independent pathways that influence the development and progression of atherosclerosis: the presence of B cells was characterized in the adventitia of atherosclerotic aortas but not in the atheromatous plaque [99]; this suggests a local immune response, associated with T cells, DC, and macrophages [100].

### 2.5. T Lymphocytes Subsets: Signalling and Mechanisms 

T cells have been found in the blood vessel walls near various CV diseases. They can contribute to immune responses in two ways: directly, by producing cytokines and molecules that promote inflammation, and indirectly through the activation of B cells. The different subsets of T cells have distinct functions in CV diseases, depending on whether they produce pro-inflammatory or anti-inflammatory molecules. In this context, we will focus on the roles of CD4+ and CD8+ T cells in atherosclerosis. CD4+ T cells, when in a naive state, can be differentiated into several subsets: T helper 1 (TH1), TH2, TH17, or regulatory T (Treg) cells. TH1 cells are pro-atherogenic and act through the production of IFN-γ and TNF-α [101,102].

On the other hand, Treg cells have an anti-atherosclerotic effect by secreting IL-10 and transforming growth factor-β (TGF-β). In fact, studies have shown that IL-10 produced by Treg cells can slow down the progression of abdominal aortic aneurysm and the formation of artery blockages following angioplasty [102,103,104,105].

TH2 cells secrete molecules such as IL-4, IL-5, and IL-13. While TH2 cells and IL-4 may be associated with advanced atherosclerosis in mice lacking the apolipoprotein E (ApoE) gene [106], atherosclerosis decreased in mice lacking both the IL4 and Ldlr or IL4 and ApoE genes [107,108]. 

Lastly, there is inconsistent contrasting evidence on the impact of TH17 cells in atherosclerosis. TH17 cells produce cytokines like IL-17A and IL-17F. Blocking or inhibiting IL-17 in mice lacking the ApoE gene was found to promote the development of atherosclerosis [109,110,111]. However, mice lacking the ILl17a gene actually showed an accelerated formation of unstable atherosclerotic lesions compared to mice lacking only the ApoE gene [112].

Assorted studies suggest that CD4+ T-cells are crucially involved in left-ventricular (LV) remodeling during both ischemic [113] and non-ischemic [114] heart failure. Mice studies show activation of CD4+ T-cells post-MI is a controlled response designed to subside rapidly with scar formation to achieve complete immune resolution within 2 weeks post-MI. HF, on the other hand, is associated with a second wave of CD4+ T-cell activation, and their transmigration into the heart promotes LV remodeling, end-diastolic volume and end-systolic volume increasing, ejection fraction reduction, and progressive cardiac dysfunction [115,116].

**Figure 2:**
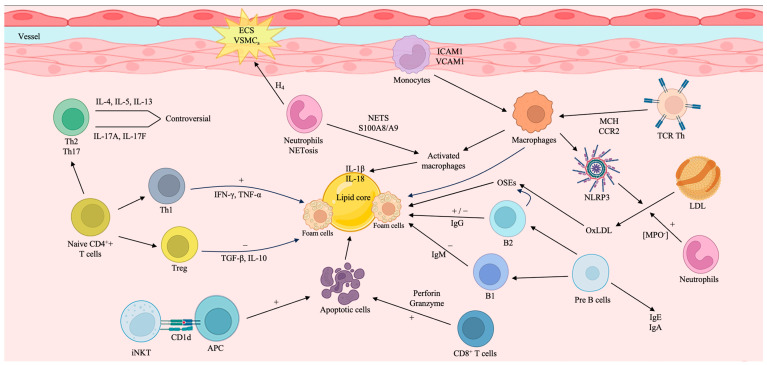
The interplay between immune cells in atherosclerotic plaque formation. The figure defines the interaction between immune cells in plaque formation, emphasizing both proatherogenic and atheroprotective mechanisms. These processes coexist with each other, so the development of atherosclerotic plaque may undergo different variations based on the release of different molecules and cytokines. APC = Antigen-Presenting Cell; B1 = B1 Cells; B2 = B2 Cells; CD1d = cluster of differentiation 1 D; EC = Endothelial Cells; H4 = Histone 4; ICAM1 = Intercellular Adhesion Molecule 1; IFN-γ = Interferon-γ; Ig = immunoglobulin; Il-4 = Interleukin-4; Il-5 = Interleukin-5; Il-10 = Interleukin-10; Il-13 = Interleukin-13; Il-17A = Interleukin-17A; Il-17F = Interleukin-17F; Il-18 = Interleukin-18; Il-1β = Interleukin-1β; iNKT = Invariant Natural Killer T Cell; LDL = Low-density Lipoprotein; MPO = Myeloperoxidase; NETs = Neutrophil Extracellular Traps; NLRP3 = nucleotide-binding oligomerisation domain-like receptor pyrin domain-containing protein 3; OSEs = Oxidation-Specific Epitopes; Ox-LDL = oxidized low-density lipoprotein; Pre-B Cells = precursors of B cells; TCR = T Cell Receptor; TGF-β = Transforming Growth Factor-β; Th = T Helper Cell; TNF-α = Tumor Necrosis Factor-α; Treg = Regulatory T Cell; VCAM1 = Vascular Cell Adhesion Molecule 1; VSMCs = Vascular Smooth Muscle Cell.

CD8+ T cells play a role in the development of atherosclerosis. When these cells are activated, they release cytotoxins, perforin, and granzymes. The cytotoxins can induce programmed cell death, or apoptosis, in macrophages, vascular smooth muscle cells (VSMCs), and ECs. This contributes to the formation of vulnerable atherosclerotic lesions, which are areas of plaque that can rupture and lead to complications [117]. Furthermore, the absence of programmed cell death ligand (PDL)-1 and PDL-2 in mice has been shown to increase the development of atherosclerotic lesions in the aorta. It also leads to an increase in the numbers of CD4+ T cells and CD8+ T cells, suggesting that these cells are more involved in atherosclerosis when these molecules are lacking [118] (Figure 2).

Moreover, T Lymphocytes crosstalk with other molecules, such as cyclophilins, have a new role in CAD: these proteins are released into the extracellular space in response to inflammatory stimuli. Gegunde et al. described the involvement of a cell surface receptor for extracellular cyclophilins in CAD, the CD147 receptor: patients with CAD had considerably higher levels of membrane expression of CD147, cyclophilin A, B, and C in T lymphocytes purified from these subjects, as well as pro-inflammatory interleukins [119]. 

## 3. Trained Immunity in Atherosclerosis: A New Proposal for a New Direction 

When briefly exposed to certain stimuli, cells of the innate immune system such as monocytes, macrophages, DC, and NKT cells can develop a phenotype resembling immunologic memory, termed trained immunity [120]. Upon restimulation, trained cells manifest a long-term proinflammatory phenotype with an increased cytokine release, nonspecific with respect to the original stimulus. The persistent overactivation of these trained cells could contribute to the incessant vascular wall inflammation, a peculiar characteristic of atherosclerosis [121]. 

Previous works on trained immunity involved microorganisms and microbial products including the Bacillus Calmette–Guerin (BCG) vaccine, *Candida albicansi,* and its cell wall component β-glucan [122].

Exposure to these pathogens provokes an increased production of proinflammatory cytokines and chemokines in trained cells as a response to a secondary insult, even if different from the initial one. 

**Figure 3:**
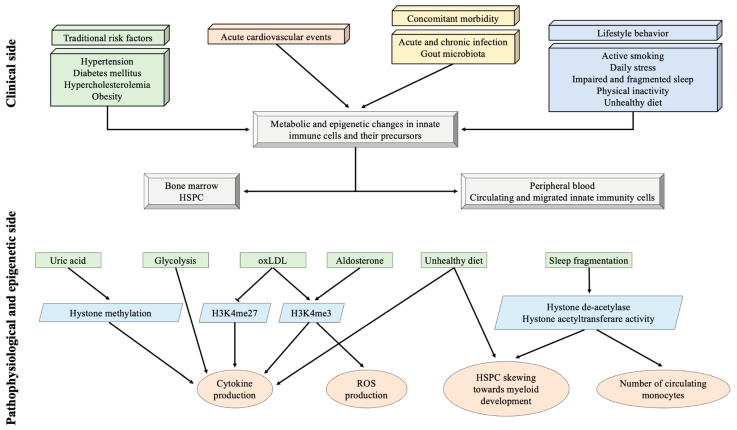
Key evidence on trained immunity. The upper side shows the clinical elements potentially determining the changes in innate immunity. The lower side reports the main pathophysiological and epigenetic mechanisms involved in the “training” of innate immune cells. H3K4me3 = histone 3 lysine 4 trimethylation; H3K27me3 = histone 3 lysine 27 trimethylation; HSPC = hematopoietic stem and progenitor cell; oxLDL = oxidized low-density lipoprotein; ROS = reactive oxygen species.

It was later recognized that also endogenous, self-derived molecules such as ox-LDL, catecholamines, uric acid, and aldosterone can induce a persistent functional reprogramming of innate immune cells [123,124,125,126,127] (Figure 3).

### 3.1. Trained Immunity in Infectious Disease 

From an evolutionary perspective, trained immunity confers protection to the host against subsequent infection, although it can also be responsible for a maladaptive state. An exogenous stimulus such as BCG vaccination protects against lethal systemic *C albicans* infection in immunodeficient mice that lack adaptive immunity [128], as well as administration of β-glucan in mice confers protection against recurrent infections [129,130]. Similar evidence also exists in humans, with the profound decrease in infant mortality rates following BCG vaccination, not solely explained by the protection against tuberculosis [131].

### 3.2. Trained Immunity in Chronic Inflammatory Disease 

Diversely from the protective effect against infections, trained immunity might be maladaptive in chronic inflammatory diseases in which innate immunity cells play a pivotal role in the pathophysiology. The detrimental effects of trained immunity are implicated in atherosclerosis, gout, neurodegenerative disorders, and transplant rejection [132,133,134], and in other inflammatory diseases such as rheumatoid arthritis and systemic lupus erythematosus [135,136].

Trained immunity could also be one of the mechanisms contributing to the epidemiological association between infectious burden and atherosclerotic CV diseases [137].

Different endogenous, nonmicrobial atherogenic stimuli have been recognized to induce trained immunity, such as ox-LDL and lipoprotein(a) (Lp(a)), but also catecholamines and high glucose concentration [124,138,139,140]. ox-LDL has a key role in atherogenic plaque formation thanks to its ability to activate immune cells and trigger foam cell formation. When exposed to a low concentration of ox-LDL and restimulated with a TLR agonist, macrophages produce higher quantities of atherogenic cytokines and chemokines, such as IL-6, MCP1 (monocyte chemoattractant protein 1), and TNF-α. Foam cell formation is similarly enhanced after exposure to ox-LDL, due to the overexpression of scavenger receptor-A (SR-A) and CD36 and downregulation of cholesterol efflux transporters adenosine triphosphate-binding cassette transporter-A1 and G1 [124,141].

Lp(a) is the main circulating carrier of oxidized phospholipids and plays a key role in atherogenesis [142,143]. Monocytes incubated with Lp(a) for 24 h show an increased proinflammatory cytokine production compared to untrained controls [144]. Monocytes isolated from patients with elevated Lp(a) levels also show a stronger trans-endothelial migration. 

A pivotal role in diabetes and CV diseases is played by diet, and atherosclerosis-prone knock-out for LDL receptor mice display characteristics of trained immunity when fed a Western-type diet [145].

The characteristic increase in cytokine production in trained immunity has also been observed in patients with already established coronary atherosclerosis, familial hypercholesterolemia, and in patients with cerebral small vessel disease [146,147]. Similarly, hyperuricemia can induce long-term proinflammatory activation of innate immune cells [123,126].

## 4. Mechanisms Involved in Trained Immunity

The trained phenotype is maintained through two key mechanisms: epigenetic and metabolic reprogramming. Both factors influence, together with the initial stimulus, the heterogeneity of the trained immune response.

### 4.1. Epigenetic Remodelling 

Epigenetic regulation is defined as the regulation of gene expression without an alteration in the DNA sequence itself. This occurs through DNA methylation, histone modifications, or post-translational modulation by non-coding RNAs. The methylation and acetylation marks regulate DNA accessibility to the transcriptional machinery. While DNA hypermethylation is usually associated with gene silencing [148], histone modifications can either lead to activation or repression of gene transcription. Histone acetylation stimulates the binding of transcription factors and activation of gene transcription [149], while the effect of histone methylation depends on the specific lysine residue involved and on the amount of methyl groups added. An in-depth review on epigenetics and trained immunity can be found elsewhere [150].

Different epigenetic marks are associated with trained immunity, the main ones being the monomethylation and trimethylation of histone 3 at lysine 4 (H3K4me1 and H3K4me3), being, respectively, a mark observed at the promoter regions of actively transcribed genes and a marker of open chromatin. H3K4me1 is typically found at enhancers and accompanied by the acetylation of histone 3 at lysine 27 (H3K27ac) [151]. 

In vitro, ox-LDL-trained macrophages show an enrichment of the activating histone modification at H3K4me3 which opens chromatin and allows transcription of genes encoding for proinflammatory and proatherogenic cytokines and chemokines such as IL6, IL-8, matrix metalloprotease (MMP)-2, MMP-9, TNF-α, SR-A, and CD36 [124]. Similar histone modifications can also be observed in trained monocytes of patients with familial hypercholesterolemia who have highly elevated levels of LDL, leading to an upregulation of immune activation, metabolic, and inflammatory pathways [152].

Lysine demethylase 5 (KDM5) histone demethylase, and SET-domain containing 7 (Set7) histone lysine methyltransferase) are other epigenetic enzymes involved in the regulation of β–glucan–induced trained immunity: KDM5 activity erase H3K4me3 marks at the promoter regions was inhibited during induction of trained immunity, while Set7 was responsible for writing H3K4me1 marks at the enhancer regions of trained immunity [153,154].

### 4.2. Metabolic Reprogramming 

The function of epigenetic enzymes can be modulated by different intermediates of metabolic pathways that act as substrates or cofactors, thus explaining the interplay between epigenetic and metabolic reprogramming in trained immunity. Three main involved metabolic pathways in trained immunity are glycolysis, glutaminolysis, and cholesterol synthesis [155]. 

Β-glucan and ox-LDL trained cells show a shift from oxidative phosphorylation to aerobic glycolysis, and this upregulation is mediated by the activation of the mammalian target of rapamycin (mTOR) pathways [59,156].

Activation of glycolysis increases, on one hand, glucose uptake with subsequent conversion to pyruvate and lactate and rapid increase in adenosine triphosphate production, and on the other it results in a high cellular NA+-to-NADH ratio, which regulates the activity of sirtuin 1 histone deacetylases. Indeed, β-glucan-induced training inhibited expression of *SIRT1* in monocytes, and the addition of the sirtuin 1 activator resveratrol partially prevented β-glucan-induced trained immunity [59]. Pyruvate is also converted into acetyl-CoA and enters the tricarboxylic acid (TCA) cycle. Several intermediates of the TCA cycle, such as fumarate, succinate, and malate accumulate in trained macrophages, and these are replenished through glutaminolysis [154]. Fumarate directly inhibits histone demethylase KDM5, which correlates with increased training and enhanced TNF-α and IL-6 production [154].

Other studies have also underlined the role of intracellular accumulation of mevalonate, a metabolite of the cholesterol synthesis process, essential for the induction of trained immunity via inducing H3K4me3 on IL-6 e TNF-α promoters [151].

Besides glycolysis, glutaminolysis, and mevalonate synthesis, oxidative phosphorylation can also induce trained immunity, through regulation of the Set7 lysine methyltransferase [153]. Meanwhile, itaconate is responsible for the balance between innate immune tolerance and trained immunity by inducing metabolic alterations in macrophages [157], and fatty acid synthesis is pivotal for trained immunity induced by the adrenal hormone aldosterone [127].

### 4.3. Clinical Applications and Future Perspectives 

The concept of trained immunity is not restricted to innate immune cells, as vascular ECs, vascular smooth muscle cells, fibroblasts, microglia, and epithelial stem cells can show long-term adaptation after brief stimulation, termed expanded trained immunity [158,159,160]. Vascular ECs act as conditional innate immune cells as they secrete cytokines and are involved in antigen presentation and phagocytosis [161], while ECs adopt a persistent inflammatory phenotype following brief exposure to high glucose concentrations [162]. Also, vascular smooth muscle cells are capable of building a sustained proinflammatory phenotype after brief exposure to ox-LDL [163].

The mechanism of trained immunity opens new avenues in research to improve prevention and treatment in a wide array of inflammatory diseases, to avoid the detrimental consequences of chronic inflammation. Therapeutic options should therefore target the well-established metabolic and epigenetic programs responsible for trained immunity. 

Several existing drugs inhibit specific metabolic pathways that drive trained immunity, including inhibitors of the mTOR pathway, hydroxymethylglutaryl-CoA inhibitors by preventing mevalonate synthesis, or NLRP3 inflammasome inhibitors [59,125]. Inhibition of the mTOR pathway prevents the increase in glycolysis and the proinflammatory phenotype in macrophages [164]. 

The specific epigenetic enzymes regulating the epigenetic reprogramming of trained cells provide another attractive pharmacological target, as several epigenetic drugs are already being adopted in hematologic and oncological disorders [165]. A nanoparticle delivery approach able to selectively target these drugs to the specific innate immune cells (e.g., plaque macrophages) can improve the specificity of these drugs and prevent off-target effects [165].

Notably, treatment with statins for three months does not revert trained immunity in vivo in familial hypercholesterolemia, while an ex vivo analysis showed that the proinflammatory phenotype in monocytes persists. Therefore, statins may prevent training, but not revert it [152]. 

An exhaustive discussion of the main therapeutic target in trained immunity has been previously published [166].

Although the detrimental effects of trained immunity have now been established, many questions remain to be answered, from identifying potential cell subsets that may be particularly amenable to trained immunity or to the relationship between trained immunity and other mechanisms of innate immune cell activation, such as clonal haematopoiesis or immune cell senescence. These aspects warrant further investigation. 

## 5. New Aspects of Endothelial and Vascular Smooth Muscle Cells

### 5.1. Endothelial Cells

The inner layer of the vessels is named endothelium, and it is formed by the ECs. ECs are known to be involved in several processes ranging from metabolic and vascular homeostasis to coagulation and permeability [161,167,168]. The secretory function of ECs includes numerous cytokines like IL-6 [169,170,171], IL-1 [172,173], placental growth factor, [174], and connective tissue growth factor [175].

A plastic role for ECs has been suggested by prior investigators in atherothrombotic disease: a potential transdifferentiation of thrombus-derived leukocytes has been proposed by Fu et al. in hypoxic conditions [176]. Moreover, ECs could be transdifferentiated from fibroblasts after the induction of innate immunity signals promoting a metabolic switch to the glycolytic substrate [177], and during atherosclerosis development the endothelium may serve as a source of plaque-associated mesenchymal cells under an endothelial-to-mesenchymal transition [178], that has been recently described occurring also in adipose tissue [179].

The above-mentioned phenomena share signaling pathways with the innate immunity, raising the hypothesis that ECs should be considered innate immune cells. 

In fact, several actions conducted by macrophages can also be performed by ECs: cytokine secretion, antigen presentation, phagocytic function, and damage-associated and pathogen-associated molecular patterns (DAMPs and PAMPs, respectively) pro- and anti-inflammatory activity [161].

Furthermore, ECs show trained immunity characteristics [180], and can support cellular homeostasis through the development of tolerance from the external stimuli, DAMPs and PAMPs [166].

The expression of known DAMPs systems by ECs enforces the evidence of being part of the innate immunity, but also the presence of receptors binding components of exogenous microbes as well as harmful endogenous components—the so-called pattern recognition receptors (PRRs)—have been proposed as novel key elements of conditional receptor of damage, features by the ability to be stimulated by endogenous metabolites and substances raising pathological concentrations, with consequent activation of inflammation [181].

The PRRs function has been suggested as a bridge between the external infection agents and/or products, and the endogenous metabolites triggering inflammation. The ECs activation mediated by ox-LDL leads to inflammation with consequent release of IL-8 through nucleotide-binding oligomerization domain (NOD)-1, and potentially cell death stage through pyroptosis mediated by caspase-1 [182]. Moreover, IL-17 can activate ECs with subsequent IL-6 release together with GM-CSF [183]. The functionally impaired endothelium due to inflammation shows a higher expression of TLR-2 and -4, similarly to the endothelium of atherosclerotic plaques [184].

Looking at adaptive immunity, several reports have underscored the EC action in influencing the action of T cells, both on the side of their activation and differentiation [185]. However, recent studies of murine models have suggested a role of the inflamed ECs in switching off the immune response: ECs activated by INF-γ and cultivated with CD4+ T cells have been found to induce the expression and polarization into immunosuppressive regulatory T cells (Treg) [186]. Furthermore, Treg activation leads to an enhanced expression of immune checkpoint receptors and increased production of the anti-inflammatory IL-10 and TGF-β [187].

### 5.2. Vascular Smooth Muscle Cells

Another crucial actor involved on the stage of atherosclerosis is represented vSMCs, which constitute the media tunica of arteries. During physiological homeostasis, vSMCs regulate blood pressure and guarantee the integrity of the vessels. vSMCs are featured by notable plasticity that allows vascular remodeling, especially in pathological conditions [188]. In the absence of damage, vSMC proliferation is markedly limited, and the specific muscle phenotype is supported. When atherosclerosis progresses, initially a positive, outward remodeling occurs to preserve the vessel patency and blood flow; however, this process can assume negative and detrimental consequences after its maximal expansion [189]. Moreover, plaque stability and integrity are related to the products and composition of the ECM, which is mostly generated by vSMCs [190].

The microenvironment laying in correspondence of the atherosclerotic lesions changes on the appropriate activity of vSMCs. Ox-LDL, pro-inflammatory cytokines like IL-8, and chemokines endorsing leucocytes recruitment and migration, accelerate the progression of the plaques. The platelet-derived growth factor-BB and TGF-β were shown to promote vSMCs differentiation, proliferation, and migration to the site of vascular plaque [191]. Conversely, the administration of IL-1β can antagonize vSMC migration and ECM production [192]; that evidence is consistent with murine models showing that anti-IL-1β antibodies decreased overall plaque burden [193].

**Figure 4:**
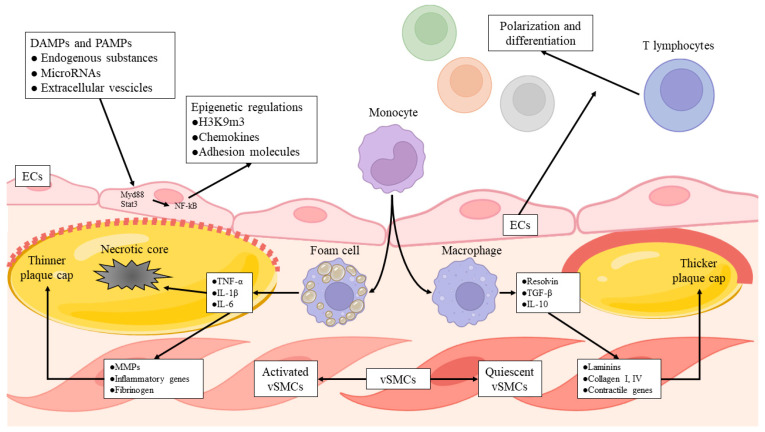
Endothelial and vascular smooth muscle cells. The figure describes the triggers and the consequent responses involving endothelial cells and vascular smooth muscle cells at the atherosclerotic plaque. These actions are configured in the context of the immune–vascular interplay. DAMPs = damage-associated molecular patterns; ECs = endothelial cells; H3K9m3 = histone modification trimethylation of lysine 9 at histone 3; IL-1β = interleukin-1β; IL-6 = interleukin-6; IL-10 = interleukin-10; MMPs = matrix metalloproteases; Myd-88 = myeloid differentiation primary response protein 88; NF-kB = nuclear factor-κB; PAMPs = pathogen associated molecular patterns; TGF-β = transforming growth factor-β; TNF-α = tumor necrosis factor-α; vSMCs = vascular smooth muscle cells.

The activity of vSMCs is strictly figured out by macrophages, particularly by their appropriate role as scavengers. In fact, in stable plaque macrophage activity, remotion of apoptotic cells is preserved [194]. That specific phagocytosis, named efferocytosis, promotes the release of IL-10, TGF-β, resolvins, and lipoxins [195], which in turn stimulate vSMCs to synthesize collagen, laminins, and other components of the ECM [196,197,198]. The vulnerability of the plaque is enhanced when macrophage efferocytosis results are impaired, with a consequent switch in cytokine secretion from pro-resolution to pro-inflammatory fashion, including TNF-α, IL-1β, and IL-6. These mediators can stimulate vSMCs through nuclear factor -kB (NF-kB) pathways [199,200], to release MMP and other inflammatory genes [201], contributing to reducing the stability of the plaque, thus aggravating the necrotic core and thinning the fibrous cap [202] (Figure 4).

## 6. Exosomes and Inflammasome

### 6.1. Exosomes

Exosomes represent a fascinating avenue of exploration within the realm of cardiovascular diseases (CVDs), with potential applications in diagnostics, therapeutics, and regenerative medicine. These tiny vesicles, secreted by various cell types including cardiomyocytes, vSMCs, ECs, and inflammatory cells, contain heat shock protein (HSP), lipids, proteins, and microRNAs [203]. These cells release exosomes containing molecules that induce or inhibit atherosclerosis, depending on the type or physiological state of the cell [204,205]. These mechanisms are key factors in regulating CVD progression, due to carrying and exchanging signaling molecules: exosomes may be able to spread atherosclerosis distally via extracellular vesicles, but also with a cell-to-cell interaction [206,207,208,209].

In recent studies, ox-LDL and homocysteine trigger the release of HSP70-containg exosomes from the aortic ECs. These exosomes activate monocytes [210,211]; once activated, they adhere to the ECs and penetrate the subendothelial space. Once inside, the activated monocytes can differentiate into macrophages, which in turn promote atherosclerotic plaque [212].

Exosomes can be engineered to transport therapeutic cargoes like small interfering RNAs (siRNAs), microRNAs, or proteins to specific cell populations involved in CVDs. This precise delivery system holds the potential to reduce inflammation, promote tissue repair, and hinder the progression of the disease [213,214].

It is important to underscore that while the application of exosomes in CVDs is a promising field, further research is essential to comprehensively understand their mechanisms, safety, and efficacy in clinical settings. 

### 6.2. The Role of Nucleotide-Binding Oligomerisation Domain-like Receptor Family Pyrin Domain Containing 3 (NLRP3) Inflammasome

The NLRP3 inflammasome is a crucial mediator of various inflammatory diseases, including atherosclerosis and other vascular diseases [215]. A wide range of different stimuli can activate NLRP3, although most of these stimuli do not directly interact with it. Among the elements that activate the NLRP3 inflammasome include a decrease in intracellular K+, due to K+ efflux, the ROS generation from mitochondria, and the release of cathepsin from lysosomes. Researchers have shown that the production of ROS from mitochondria is crucial for activating the NLRP3 inflammasome: studies suggest that oxidized mitochondrial DNA released in response to NLRP3 activators can drive its activation. Additionally, the release of cathepsin from damaged lysosomes can trigger its activation [216]. Several studies have shown that the expression levels of NLRP3 inflammasome components may play a role in the progression of atherosclerosis. For instance, Zeng et al. used a selective inhibitor of the NLRP3 inflammasome called MCC950 in ApoE^−/−^ mice to inhibit its activity by reducing the pyroptosis of macrophages and IL-1β and IL-18 production in the aorta and in cell lysates. Instead of interfering with the NLRP3 inflammasome’s priming, MCC950’s anti-atherosclerotic actions on reducing macrophage inflammation and pyroptosis required limiting the assembly and activation of the inflammasome [217]. These studies have supplied direct evidence that the NLRP3 inflammasome contributes to the progression of atherosclerosis, therefore targeting the NLRP3 inflammasome could potentially be a therapeutic strategy for treating atherosclerosis. However, there are some conflicting data about the role of the NLRP3 inflammasome in atherosclerosis [218]. Recent research by Chen et al. has reported that the lack of NLRP3 in bone marrow cells specifically attenuated the formation of atherosclerotic lesions in LDL receptor^−/−^ females. However, there was no significant effect observed in male mice [219]. Despite promising evidence, NLRP3 inflammasome deserves further studies to answer all the grey issues about it.

## 7. Therapeutic Strategies—New Perspectives

### 7.1. Mineralocorticoid Antagonists

The receptor for mineralocorticoid (MR) is included among the family of nuclear receptor steroid binding. Besides the original indication of MR antagonists (MRA) in primary aldosteronism, MRA showed substantial benefits in the CV field, mostly in heart failure [220,221].

The protective action of MRA may be explained by the wide expression of MR not only on renal cells: it was found also in ECs, leading to aldosterone-dependent expression of vascular cells and intercellular adhesion molecules [222,223], and also in the cells of the innate immune system [224]. 

Evidence suggests a role for aldosterone and, conversely, its antagonist, spironolactone, in the polarization of macrophage phenotypes. In rats, M1 macrophages were shown to express a higher number of CD68 in tissue, coupled with an increased production of TNF-α and reactive oxygen species after injection of aldosterone [225]. As expected, the administration of spironolactone reduces the expression of M1 markers [226]. In dedicated murine model knockout for MR, protection against cardiac remodeling and myocardium macrophage M1 infiltration was observed [227], as well as a reduction in M1-like phenotype and an increase of M2 phenotype [226].

Some reports have investigated the role of ox-LDL intake by macrophage, underscoring an enhanced lipid intake and foam cell formation in upregulated renin-angiotensin-aldosterone system models [228], together with a potential increase in the expression of genes related to cholesterol efflux by macrophages in model knockout for MR [229]. 

### 7.2. Role of Interleukin-2

IL-2 is a common gamma chain cytokine that plays a crucial role in maintaining Treg homeostasis [230]. IL-2 is critical for the sustenance of Foxp3+ Treg, and its absence leads to a significant shortage of Treg cells, resulting in autoimmunity [231]. IL-2 has a co-stimulatory role over group 2 innate lymphoid cells (ILC2) and is located in primary immune organs like the spleen and bone marrow, as well as within tissues such as the lung, gut, and adipose tissue [232,233]. ILC2 are recognized for releasing significant amounts of type II cytokines (such as IL-5, IL-13, IL-9), governing both innate and adaptive immune responses across various inflammatory scenarios; additionally, they also play a role in wound healing and tissue repair modulation, while impacting the function of adipose tissue and metabolic equilibrium [234,235,236,237]. The secretion of IL-5 and IL-13 by ILC2 is linked to beneficial atheroprotective B1b-cells and tissue repair programs [238]. 

IL-2 administration has been shown to increase CD4+CD25+Foxp3+ Treg numbers in patients with cancer [239]. Rosenzwajg et al. evaluated the effect of low-dose IL-2 across eleven autoimmune diseases: in their phase I trial, they reported an activation and expansion of Treg cells [240]. On the atherosclerotic field, the phase I/II clinical trial “LILACS study” has the aim to evaluate the potential role of low-dose IL-2, aldesleukin, in patients affected by stable ischemic heart disease and acute coronary syndromes: primary endpoints are related to the safety and tolerability of aldesleukin, and in one of the exploratory analyses, the effect of ld-IL-2 on ILC2s and its correlation with vascular inflammation [241]. 

### 7.3. Checkpoints Inhibitors

Immune-checkpoint proteins are membrane proteins expressed on APC and T-cells, and their interaction leads to T-cell activation. However, immune checkpoint proteins also have a pivotal role in mediating interactions between immune cells and non-immune cells, and these interplays regulate different pathways, such as secretion of cytokines and chemokines, cellular survival, and proliferation, thus collectively shaping the inflammatory response [242]. 

Currently, oncology is the main field of application of so-called immune-checkpoint inhibitors, with cytotoxic T-lymphocyte antigen 4 (CTLA-4), programmed cell death protein (PD)-1, and PDL-1 as the main immune-checkpoint proteins involved [243]. In recent years, these drugs have transitioned from advanced and metastatic settings to a (neo)adjuvant one, carving out a role in the treatment of potentially curable patients [244]. However, these treatments are burdened by important side effects, especially endocrine (e.g., thyroid and adrenal dysfunction), gastrointestinal (diarrhoea), pulmonary (pneumonitis), dermatological (dermatitis, psoriasis), and CV (myocarditis) toxicities [245]. 

Because of the binding between CV disease and immune-checkpoint pathways, and the role of inflammation in developing atherothrombosis, recent studies are trying to evaluate a potential role of modulating this immune signaling. The CANTOS trial evaluated the use of Canakinumab, interleukin-1β blocker, in 10,061 patients with previous MI and a high-sensitivity C-reactive protein level. The patients were randomized to receive Canakinumab versus placebo, and those treated with the first one at a subcutaneous dose of 150 mg once every 3 months had a noticeably reduced occurrence of recurring CV events [246]. Pre-clinical studies have evaluated the interaction between CTLA-4 and B7-1/B7-2 as an atheroprotective effect. Mice treated with CTLA-4 analogue (abatacept) had a reduction in femoral arteries atherosclerosis formation by 78%, also in murine models with a decreased expression of CTLA-4 membrane expression [247,248]; on the contrary, anti-CTLA-4 drugs are related to a plaque progression [249]. Considering the PD-1, PDL-1 pathway, different studies have noted reduced PD-1 expression or its ligands in individuals with CAD and acute coronary syndrome, implying its protective role in atherogenesis and the development of an advanced plaque phenotype [250,251].

## 8. Future Direction and Conclusions

CAD is a complex condition characterized by the buildup of plaque in the arteries that supply blood to the heart. Chronic inflammation plays a significant role in the development and progression of CAD. It is important to explore ways to modulate the immune response to reduce inflammation in the arterial walls. This includes studying the use of anti-inflammatory medications, such as IL-1β inhibitors (i.e., Canakinumab), or exogenous IL-2 administration, to reduce the risk of cardiovascular events in individuals with CAD. Immune cells, particularly macrophages and T lymphocytes, are critical in the formation and destabilization of atherosclerotic plaques. Understanding novel immune mechanisms involved can lead to targeted therapies aimed at modulating immune cell activity in the context of CAD. Identifying specific immune biomarkers that can predict CAD risk or disease progression is an area of active investigation. These biomarkers could help in early detection and risk assessment. It is crucial to the potential of immunomodulatory therapies, for example, monoclonal antibodies, to reduce inflammation and plaque formation in CAD. Trained immunity could be a new therapeutic aim, opening up new treatment and prevention of inflammatory diseases, and avoiding chronic inflammation. These options should target the well-established metabolic and epigenetic programs responsible for trained immunity. The gut microbiome has recently emerged as another area of interest in CAD research. There is evidence to suggest that the composition of gut bacteria may influence systemic inflammation and immune responses, which could impact the CAD risk. Understanding these interactions may lead to novel interventions. Tailoring CAD treatments based on an individual’s immune profile is an emerging concept: personalized medicine approaches may involve identifying a patient’s specific immune-related risk factors and customizing treatment strategies accordingly. For instance, the use of exosome-based therapies can be tailored to an individual’s specific requirements. Analyzing a patient’s exosomes may enable the development of personalized treatment strategies, optimizing the effectiveness of interventions in managing CVDs, especially in CAD.

Future CAD treatments may involve combining traditional approaches, such as statins and antiplatelet drugs, with immunomodulatory agents to achieve better outcomes. It is important to note that while inflammation and the immune system take part in the development and complications of CAD, they are just one piece of the puzzle. CAD is a multifactorial disease influenced by genetics, lifestyle, and other risk factors.

## Data Availability

No new data were created or analyzed in this study. Data sharing is not applicable to this review.

## Abbreviations

APC	Antigen-Presenting Cell
ApoE	Apolipoprotein E
BCG	Bacillus Calmette-Guérin
CAD	Coronary Artery Disease
CCR2	C-C chemokine receptor type 2
CD	Cluster of Differentiation
CTGF	Connective Tissue Growth Factor
CTLA-4	Cytotoxic T-Lymphocyte Antigen 4
CV	Cardiovascular
CVD	Cardiovascular Disease
DAMP	Damage-Associated Molecular Patterns
DNA	Deoxyribonucleic acid
ECs	Endothelial Cells
ECM	Extracellular Matrix
GM-CSF	Granulocyte-Macrophage Colony Stimulating Factor
H3K4me1	Monomethylation of Histone 3 at Lysine 4
H3K4me3	Trimethylation of Histone 3 at Lysine 4
H3K27ac	Acetylation of Histone 3 at Lysine 27
HSP	Heat Shock Protein
IFN-γ	Interferon-γ
IL	Interleukin
ILC2	Group 2 Innate Lymphoid Cell
iNKT	Invariant Natural Killer T Cell
LP(a)	Lipoprotein A
LDL	Low-Density Lipoprotein
LDLR	Low-Density Lipoprotein Receptor
LV	Left Ventricle
MCP1	Monocyte Chemoattractant Protein 1
MHC	Major Histocompatibility Complex
MI	Myocardial Infarction
MMP	Matrix Metalloproteinase
MR	Mineralcorticoid
mTOR	Mammalian Target of Rapamycin
NET	Neutrophil Extracellular Traps
NF-kB	Nuclear factor kappa-light-chain-enhancer of activated B cells
NKT	Natural Killer T Cell
NLRP3	Nucleotide-binding Oligomerisation domain-like Receptor Pyrin Domain-containing Protein 3
NOD	Nucleotide-binding Oligomerization Domain
OSE	Oxidation-Specific Epitopes
Ox-LDL	Oxidized Low-Density Lipoprotein
PAMP	Pathogen-Associated Molecular Patterns
PD	Programmed Cell Death Protein
PDGF	Platelet-Derived Growth Factor
PDL	Programmed Cell Death Ligand
PRR	Pattern Recognition Receptor
RNA	Ribonucleic Acid
SR-A	Scavenger Receptor-A
siRNA	Small Interfering RNA
TCA	Tricarboxylic Acid
TCR	T Cell Receptor
TGF-β	Transforming Growth Factor
TLR	Toll-Like Receptor
TNF-α	Tumor Necrosis Factor-α
vSMCs	Vascular Smooth Muscle Cell

## References

[1] Libby P. (2021). The Changing Landscape of Atherosclerosis. Nature.

[2] Libby P. (2012). Inflammation in Atherosclerosis. Arter. Thromb. Vasc. Biol..

[3] Hansson G.K. (2005). Inflammation, Atherosclerosis, and Coronary Artery Disease. N. Engl. J. Med..

[4] Sreejit G., Johnson J., Jaggers R.M., Dahdah A., Murphy A.J., Hanssen N.M.J., Nagareddy P.R. (2022). Neutrophils in Cardiovascular Disease: Warmongers, Peacemakers, or Both?. Cardiovasc. Res..

[5] Lavine K.J., Pinto A.R., Epelman S., Kopecky B.J., Clemente-Casares X., Godwin J., Rosenthal N., Kovacic J.C. (2018). The Macrophage in Cardiac Homeostasis and Disease: JACC Macrophage in CVD Series (Part 4). J. Am. Coll. Cardiol..

[6] Simons K.H., de Jong A., Jukema J.W., de Vries M.R., Arens R., Quax P.H.A. (2019). T Cell Co-Stimulation and Co-Inhibition in Cardiovascular Disease: A Double-Edged Sword. Nat. Rev. Cardiol..

[7] Porsch F., Mallat Z., Binder C.J. (2021). Humoral Immunity in Atherosclerosis and Myocardial Infarction: From B Cells to Antibodies. Cardiovasc. Res..

[8] Engelen S.E., Robinson A.J.B., Zurke Y.X., Monaco C. (2022). Therapeutic Strategies Targeting Inflammation and Immunity in Atherosclerosis: How to Proceed?. Nat. Rev. Cardiol..

[9] Ley K., Hoffman H.M., Kubes P., Cassatella M.A., Zychlinsky A., Hedrick C.C., Catz S.D. (2018). Neutrophils: New Insights and Open Questions. Sci. Immunol..

[10] Silvestre-Roig C., Braster Q., Wichapong K., Lee E.Y., Teulon J.M., Berrebeh N., Winter J., Adrover J.M., Santos G.S., Froese A. (2019). Externalized Histone H4 Orchestrates Chronic Inflammation by Inducing Lytic Cell Death. Nature.

[11] Joseph J.P., Reyes E., Guzman J., O’Doherty J., McConkey H., Arri S., Kakkar R., Beckley N., Douiri A., Barrington S.F. (2017). CXCR2 Inhibition—A Novel Approach to Treating CoronAry Heart DiseAse (CICADA): Study Protocol for a Randomised Controlled Trial. Trials.

[12] Chia S., Nagurney J.T., Brown D.F.M., Raffel O.C., Bamberg F., Senatore F., Wackers F.J.T., Jang I.K. (2009). Association of Leukocyte and Neutrophil Counts with Infarct Size, Left Ventricular Function and Outcomes after Percutaneous Coronary Intervention for ST-Elevation Myocardial Infarction. Am. J. Cardiol..

[13] Guasti L., Dentali F., Castiglioni L., Maroni L., Marino F., Squizzato A., Ageno W., Gianni M., Gaudio G., Grandi A.M. (2011). Neutrophils and Clinical Outcomes in Patients with Acute Coronary Syndromes and/or Cardiac Revascularization: A Systematic Review on More than 34,000 Subjects. Thromb. Haemost..

[14] Verdoia M., Nardin M., Gioscia R., Negro F., Marcolongo M., Suryapranata H., Kedhi E., De Luca G. (2020). Higher Neutrophil-to-Lymphocyte Ratio (NLR) Increases the Risk of Suboptimal Platelet Inhibition and Major Cardiovascular Ischemic Events among ACS Patients Receiving Dual Antiplatelet Therapy with Ticagrelor. Vasc. Pharmacol..

[15] Prabhu S.D., Frangogiannis N.G. (2016). The Biological Basis for Cardiac Repair after Myocardial Infarction. Circ. Res..

[16] Sreejit G., Abdel Latif A., Murphy A.J., Nagareddy P.R. (2020). Emerging Roles of Neutrophil-Borne S100A8/A9 in Cardiovascular Inflammation. Pharmacol. Res..

[17] Ortega-Gomez A., Salvermoser M., Rossaint J., Pick R., Brauner J., Lemnitzer P., Tilgner J., De Jong R.J., Megens R.T.A., Jamasbi J. (2016). Cathepsin G Controls Arterial but Not Venular Myeloid Cell Recruitment. Circulation.

[18] Zarbock A., Ley K. (2008). Mechanisms and Consequences of Neutrophil Interaction with the Endothelium. Am. J. Pathol..

[19] Rasmuson J., Kenne E., Wahlgren M., Soehnlein O., Lindbom L. (2019). Heparinoid Sevuparin Inhibits Streptococcus-Induced Vascular Leak through Neutralizing Neutrophil-Derived Proteins. FASEB J..

[20] Mawhin M.-A., Tilly P., Zirka G., Charles A.-L., Slimani F., Vonesch J.-L., Michel J.-B., Bäck M., Norel X., Fabre J.-E. (2018). Neutrophils Recruited by Leukotriene B4 Induce Features of Plaque Destabilization during Endotoxaemia. Cardiovasc. Res..

[21] Arruda-Olson A.M., Reeder G.S., Bell M.R., Weston S.A., Roger V.L. (2009). Neutrophilia Predicts Death and Heart Failure after Myocardial Infarction: A Community-Based Study. Circ. Cardiovasc. Qual. Outcomes.

[22] Sreejit G., Abdel-Latif A., Athmanathan B., Annabathula R., Dhyani A., Noothi S.K., Quaife-Ryan G.A., Al-Sharea A., Pernes G., Dragoljevic D. (2020). Neutrophil-Derived S100A8/A9 Amplify Granulopoiesis after Myocardial Infarction. Circulation.

[23] Nagareddy P.R., Sreejit G., Abo-Aly M., Jaggers R.M., Chelvarajan L., Johnson J., Pernes G., Athmanathan B., Abdel-Latif A., Murphy A.J. (2020). NETosis Is Required for S100A8/A9-Induced Granulopoiesis after Myocardial Infarction. Arter. Thromb. Vasc. Biol..

[24] Vasilyev N., Williams T., Brennan M.L., Unzek S., Zhou X., Heinecke J.W., Spitz D.R., Topol E.J., Hazen S.L., Pen M.S. (2005). Myeloperoxidase-Generated Oxidants Modulate Left Ventricular Remodeling but Not Infarct Size after Myocardial Infarction. Circulation.

[25] Marinković G., Koenis D.S., De Camp L., Jablonowski R., Graber N., De Waard V., De Vries C.J., Goncalves I., Nilsson J., Jovinge S. (2020). S100A9 Links Inflammation and Repair in Myocardial Infarction. Circ. Res..

[26] Ma Y., Yabluchanskiy A., Iyer R.P., Cannon P.L., Flynn E.R., Jung M., Henry J., Cates C.A., Deleon-Pennell K.Y., Lindsey M.L. (2016). Temporal Neutrophil Polarization Following Myocardial Infarction. Cardiovasc. Res..

[27] Lörchner H., Pöling J., Gajawada P., Hou Y., Polyakova V., Kostin S., Adrian-Segarra J.M., Boettger T., Wietelmann A., Warnecke H. (2015). Myocardial Healing Requires Reg3β-Dependent Accumulation of Macrophages in the Ischemic Heart. Nat. Med..

[28] Massena S., Christoffersson G., Vågesjö E., Seignez C., Gustafsson K., Binet F., Hidalgo C.H., Giraud A., Lomei J., Weström S. (2015). Identification and Characterization of VEGF-A-Responsive Neutrophils Expressing CD49d, VEGFR1, and CXCR4 in Mice and Humans. Blood.

[29] Wang J., Hossain M., Thanabalasuriar A., Gunzer M., Meininger C., Kubes P. (2017). Visualizing the Function and Fate of Neutrophils in Sterile Injury and Repair. Science.

[30] García-Prieto J., Villena-Gutiérrez R., Gómez M., Bernardo E., Pun-García A., García-Lunar I., Crainiciuc G., Fernández-Jiménez R., Sreeramkumar V., Bourio-Martínez R. (2017). Neutrophil Stunning by Metoprolol Reduces Infarct Size. Nat. Commun..

[31] Marinković G., Grauen Larsen H., Yndigegn T., Szabo I.A., Mares R.G., De Camp L., Weiland M., Tomas L., Goncalves I., Nilsson J. (2019). Inhibition of Pro-Inflammatory Myeloid Cell Responses by Short-Term S100A9 Blockade Improves Cardiac Function after Myocardial Infarction. Eur. Heart J..

[32] Horckmans M., Ring L., Duchene J., Santovito D., Schloss M.J., Drechsler M., Weber C., Soehnlein O., Steffens S. (2017). Neutrophils Orchestrate Post-Myocardial Infarction Healing by Polarizing Macrophages towards a Reparative Phenotype. Eur. Heart J..

[33] Ma Y., Mouton A.J., Lindsey M.L. (2018). Cardiac Macrophage Biology in the Steady-State Heart, the Aging Heart, and Following Myocardial Infarction. Transl. Res..

[34] Heidt T., Courties G., Dutta P., Sager H.B., Sebas M., Iwamoto Y., Sun Y., Da Silva N., Panizzi P., Van Der Lahn A.M. (2014). Differential Contribution of Monocytes to Heart Macrophages in Steady-State and after Myocardial Infarction. Circ. Res..

[35] Glaros T. (2009). Macrophages and Fibroblasts during Inflammation, Tissue Damage and Organ Injury. Front. Biosci..

[36] Frantz S., Nahrendorf M. (2014). Cardiac Macrophages and Their Role in Ischaemic Heart Disease. Cardiovasc. Res..

[37] Nahrendorf M., Swirski F.K., Aikawa E., Stangenberg L., Wurdinger T., Figueiredo J.L., Libby P., Weissleder R., Pittet M.J. (2007). The Healing Myocardium Sequentially Mobilizes Two Monocyte Subsets with Divergent and Complementary Functions. J. Exp. Med..

[38] Nahrendorf M., Swirski F.K. (2016). Abandoning M1/M2 for a Network Model of Macrophage Function. Circ. Res..

[39] Xue J., Schmidt S.V., Sander J., Draffehn A., Krebs W., Quester I., DeNardo D., Gohel T.D., Emde M., Schmidleithner L. (2014). Transcriptome-Based Network Analysis Reveals a Spectrum Model of Human Macrophage Activation. Immunity.

[40] Cochain C., Vafadarnejad E., Arampatzi P., Pelisek J., Winkels H., Ley K., Wolf D., Saliba A.E., Zernecke A. (2018). Single-Cell RNA-Seq Reveals the Transcriptional Landscape and Heterogeneity of Aortic Macrophages in Murine Atherosclerosis. Circ. Res..

[41] DeBerge M., Yeap X.Y., Dehn S., Zhang S., Grigoryeva L., Misener S., Procissi D., Zhou X., Lee D.C., Muller W.A. (2017). MerTK Cleavage on Resident Cardiac Macrophages Compromises Repair after Myocardial Ischemia Reperfusion Injury. Circ. Res..

[42] Epelman S., Lavine K.J., Beaudin A.E., Sojka D.K., Carrero J.A., Calderon B., Brija T., Gautier E.L., Ivanov S., Satpathy A.T. (2014). Embryonic and Adult-Derived Resident Cardiac Macrophages Are Maintained through Distinct Mechanisms at Steady State and during Inflammation. Immunity.

[43] Epelman S., Lavine K.J., Randolph G.J. (2014). Origin and Functions of Tissue Macrophages. Immunity.

[44] Li W., Hsiao H., Higashikubo R., Saunders B.T., Bharat A., Goldstein D.R., Krupnick A.S., Gelman A.E., Lavine K.J., Kreisel D. (2016). Heart-Resident CCR2^+^ Macrophages Promote Neutrophil Extravasation through TLR9/MyD88/CXCL5 Signaling. JCI Insight.

[45] Jung K., Kim P., Leuschner F., Gorbatov R., Kim J.K., Ueno T., Nahrendorf M., Yun S.H. (2013). Endoscopic Time-Lapse Imaging of Immune Cells in Infarcted Mouse Hearts. Circ. Res..

[46] Sager H.B., Hulsmans M., Lavine K.J., Moreira M.B., Heidt T., Courties G., Sun Y., Iwamoto Y., Tricot B., Khan O.F. (2016). Proliferation and Recruitment Contribute to Myocardial Macrophage Expansion in Chronic Heart Failure. Circ. Res..

[47] Hilgendorf I., Gerhardt L.M.S., Tan T.C., Winter C., Holderried T.A.W., Chousterman B.G., Iwamoto Y., Liao R., Zirlik A., Scherer-Crosbie M. (2014). Ly-6 Chigh Monocytes Depend on Nr4a1 to Balance Both Inflammatory and Reparative Phases in the Infarcted Myocardium. Circ. Res..

[48] Christia P., Bujak M., Gonzalez-Quesada C., Chen W., Dobaczewski M., Reddy A., Frangogiannis N.G. (2013). Systematic Characterization of Myocardial Inflammation, Repair, and Remodeling in a Mouse Model of Reperfused Myocardial Infarction. J. Histochem. Cytochem..

[49] Jung M., Ma Y., Iyer R.P., DeLeon-Pennell K.Y., Yabluchanskiy A., Garrett M.R., Lindsey M.L. (2017). IL-10 Improves Cardiac Remodeling after Myocardial Infarction by Stimulating M2 Macrophage Polarization and Fibroblast Activation. Basic Res. Cardiol..

[50] Godwin J., Kuraitis D., Rosenthal N. (2014). Extracellular Matrix Considerations for Scar-Free Repair and Regeneration: Insights from Regenerative Diversity among Vertebrates. Int. J. Biochem. Cell Biol..

[51] Mescher A.L. (2017). Macrophages and Fibroblasts during Inflammation and Tissue Repair in Models of Organ Regeneration. Regeneration.

[52] Kuznetsova T., Prange K.H.M., Glass C.K., de Winther M.P.J. (2020). Transcriptional and Epigenetic Regulation of Macrophages in Atherosclerosis. Nat. Rev. Cardiol..

[53] Piccolo V., Curina A., Genua M., Ghisletti S., Simonatto M., Sabò A., Amati B., Ostuni R., Natoli G. (2017). Opposing Macrophage Polarization Programs Show Extensive Epigenomic and Transcriptional Cross-Talk. Nat. Immunol..

[54] Czimmerer Z., Daniel B., Horvath A., Rückerl D., Nagy G., Kiss M., Peloquin M., Budai M.M., Cuaranta-Monroy I., Simandi Z. (2018). The Transcription Factor STAT6 Mediates Direct Repression of Inflammatory Enhancers and Limits Activation of Alternatively Polarized Macrophages. Immunity.

[55] Li Z., Martin M., Zhang J., Huang H.Y., Bai L., Zhang J., Kang J., He M., Li J., Maurya M.R. (2017). Krüppel-like Factor 4 Regulation of Cholesterol-25-Hydroxylase and Liver X Receptor Mitigates Atherosclerosis Susceptibility. Circulation.

[56] Ostuni R., Piccolo V., Barozzi I., Polletti S., Termanini A., Bonifacio S., Curina A., Prosperini E., Ghisletti S., Natoli G. (2013). Latent Enhancers Activated by Stimulation in Differentiated Cells. Cell.

[57] Novakovic B., Habibi E., Wang S.Y., Arts R.J.W., Davar R., Megchelenbrink W., Kim B., Kuznetsova T., Kox M., Zwaag J. (2016). β-Glucan Reverses the Epigenetic State of LPS-Induced Immunological Tolerance. Cell.

[58] Foster S.L., Hargreaves D.C., Medzhitov R. (2007). Gene-Specific Control of Inflammation by TLR-Induced Chromatin Modifications. Nature.

[59] Cheng S.C., Quintin J., Cramer R.A., Shepardson K.M., Saeed S., Kumar V., Giamarellos-Bourboulis E.J., Martens J.H.A., Rao N.A., Aghajanirefah A. (2014). MTOR- and HIF-1α-Mediated Aerobic Glycolysis as Metabolic Basis for Trained Immunity. Science.

[60] Getz G.S., Reardon C.A. (2017). Natural Killer T Cells in Atherosclerosis. Nat. Rev. Cardiol..

[61] Liao C.M., Zimmer M.I., Wang C.R. (2013). The Functions of Type I and Type II Natural Killer T Cells in Inflammatory Bowel Diseases. Inflamm. Bowel Dis..

[62] Constantinides M.G., Bendelac A. (2013). Transcriptional Regulation of the NKT Cell Lineage. Curr. Opin. Immunol..

[63] Anderson B.L., Teyton L., Bendelac A., Savage P.B. (2013). Stimulation of Natural Killer T Cells by Glycolipids. Molecules.

[64] Hansson G.K., Hermansson A. (2011). The Immune System in Atherosclerosis. Nat. Immunol..

[65] Yanaba K., Bouaziz J.D., Haas K.M., Poe J.C., Fujimoto M., Tedder T.F. (2008). A Regulatory B Cell Subset with a Unique CD1d^hi^CD5^+^ Phenotype Controls T Cell-Dependent Inflammatory Responses. Immunity.

[66] Tupin E., Kinjo Y., Kronenberg M. (2007). The Unique Role of Natural Killer T Cells in the Response to Microorganisms. Nat. Rev. Microbiol..

[67] Van Kaer L., Parekh V.V., Wu L. (2011). Invariant Natural Killer T Cells: Bridging Innate and Adaptive Immunity. Cell Tissue Res..

[68] Ait-Oufella H., Taleb S., Mallat Z., Tedgui A. (2011). Recent Advances on the Role of Cytokines in Atherosclerosis. Arter. Thromb. Vasc. Biol..

[69] Kleemann R., Zadelaar S., Kooistra T. (2008). Cytokines and Atherosclerosis: A Comprehensive Review of Studies in Mice. Cardiovasc. Res..

[70] Monteiro M., Almeida C.F., Caridade M., Ribot J.C., Duarte J., Agua-Doce A., Wollenberg I., Silva-Santos B., Graca L. (2010). Identification of Regulatory Foxp3^+^ Invariant NKT Cells Induced by TGF-β. J. Immunol..

[71] Choi S.H., Sviridov D., Miller Y.I. (2017). Oxidized Cholesteryl Esters and Inflammation. Biochim. Biophys. Acta Mol. Cell Biol. Lipids.

[72] Curtiss L.K., Tobias P.S. (2009). Emerging Role of Toll-like Receptors in Atherosclerosis. J. Lipid Res..

[73] VanderLaan P.A., Reardon C.A., Sagiv Y., Blachowicz L., Lukens J., Nissenbaum M., Wang C.R., Getz G.S. (2007). Characterization of the Natural Killer T-Cell Response in an Adoptive Transfer Model of Atherosclerosis. Am. J. Pathol..

[74] Nakai Y., Iwabuchi K., Fujii S., Ishimori N., Dashtsoodol N., Watano K., Mishima T., Iwabuchi C., Tanaka S., Bezbradica J.S. (2004). Natural Killer T Cells Accelerate Atherogenesis in Mice. Blood.

[75] Upadhye A., Srikakulapu P., Gonen A., Hendrikx S., Perry H.M., Nguyen A., McSkimming C., Marshall M.A., Garmey J.C., Taylor A.M. (2019). Diversification and CXCR4-Dependent Establishment of the Bone Marrow B-1a Cell Pool Governs Atheroprotective IgM Production Linked to Human Coronary Atherosclerosis. Circ. Res..

[76] Prohaska T.A., Que X., Diehl C.J., Hendrikx S., Chang M.W., Jepsen K., Glass C.K., Benner C., Witztum J.L. (2018). Massively Parallel Sequencing of Peritoneal and Splenic B Cell Repertoires Highlights Unique Properties of B-1 Cell Antibodies. J. Immunol..

[77] Baumgarth N. (2016). B-1 Cell Heterogeneity and the Regulation of Natural and Antigen-Induced IgM Production. Front. Immunol..

[78] Farias-Itao D.S., Pasqualucci C.A., Nishizawa A., da Silva L.F.F., Campos F.M., Bittencourt M.S., da Silva K.C.S., Leite R.E.P., Grinberg L.T., Ferretti-Rebustini R.E.d.L. (2019). B Lymphocytes and Macrophages in the Perivascular Adipose Tissue Are Associated with Coronary Atherosclerosis: An Autopsy Study. J. Am. Heart Assoc..

[79] Kyaw T., Tay C., Krishnamurthi S., Kanellakis P., Agrotis A., Tipping P., Bobik A., Toh B.H. (2011). B1a B Lymphocytes Are Atheroprotective by Secreting Natural IgM That Increases IgM Deposits and Reduces Necrotic Cores in Atherosclerotic Lesions. Circ. Res..

[80] Pattarabanjird T., Li C., McNamara C. (2021). B Cells in Atherosclerosis: Mechanisms and Potential Clinical Applications. JACC Basic Transl. Sci..

[81] Adamo L., Rocha-Resende C., Mann D.L. (2020). The Emerging Role of B Lymphocytes in Cardiovascular Disease. Annu. Rev. Immunol..

[82] Sage A.P., Tsiantoulas D., Binder C.J., Mallat Z. (2019). The Role of B Cells in Atherosclerosis. Nat. Rev. Cardiol..

[83] Kyaw T., Tay C., Khan A., Dumouchel V., Cao A., To K., Kehry M., Dunn R., Agrotis A., Tipping P. (2010). Conventional B2 B Cell Depletion Ameliorates Whereas Its Adoptive Transfer Aggravates Atherosclerosis. J. Immunol..

[84] Ait-Oufella H., Herbin O., Bouaziz J.D., Binder C.J., Uyttenhove C., Laurans L., Taleb S., Van Vré E., Esposito B., Vilar J. (2010). B Cell Depletion Reduces the Development of Atherosclerosis in Mice. J. Exp. Med..

[85] Grasset E.K., Duhlin A., Agardh H.E., Ovchinnikova O., Hägglöf T., Forsell M.N., Paulsson-Berne G., Hansson G.K., Ketelhuth D.F.J., Karlsson M.C.I. (2015). Sterile Inflammation in the Spleen during Atherosclerosis Provides Oxidation-Specific Epitopes That Induce a Protective B-Cell Response. Proc. Natl. Acad. Sci. USA.

[86] Friedman P., Hörkkö S., Steinberg D., Witztum J.L., Dennis E.A. (2002). Correlation of Antiphospholipid Antibody Recognition with the Structure of Synthetic Oxidized Phospholipids. Importance of Schiff Base Formation and Aldol Condensation. J. Biol. Chem..

[87] Boullier A., Gillotte K.L., Hörkkö S., Green S.R., Friedman P., Dennis E.A., Witztum J.L., Steinberg D., Quehenberger O. (2000). The Binding of Oxidized Low Density Lipoprotein to Mouse CD36 Is Mediated in Part by Oxidized Phospholipids That Are Associated with Both the Lipid and Protein Moieties of the Lipoprotein. J. Biol. Chem..

[88] Palinski W., Hörkkö S., Miller E., Steinbrecher U.P., Powell H.C., Curtiss L.K., Witztum J.L. (1996). Cloning of Monoclonal Autoantibodies to Epitopes of Oxidized Lipoproteins from Apolipoprotein E-Deficient Mice: Demonstration of Epitopes of Oxidized Low Density Lipoprotein in Human Plasma. J. Clin. Investig..

[89] Yla-Herttuala S., Palinski W., Butler S.W., Picard S., Steinberg D., Witztum J.L. (1994). Rabbit and Human Atherosclerotic Lesions Contain IgG That Recognizes Epitopes of Oxidized LDL. Arterioscler. Thromb..

[90] Chou M.Y., Fogelstrand L., Hartvigsen K., Hansen L.F., Woelkers D., Shaw P.X., Choi J., Perkmann T., Bäckhed F., Miller Y.I. (2009). Oxidation-Specific Epitopes Are Dominant Targets of Innate Natural Antibodies in Mice and Humans. J. Clin. Investig..

[91] Imai Y., Kuba K., Neely G.G., Yaghubian-Malhami R., Perkmann T., van Loo G., Ermolaeva M., Veldhuizen R., Leung Y.H.C., Wang H. (2008). Identification of Oxidative Stress and Toll-like Receptor 4 Signaling as a Key Pathway of Acute Lung Injury. Cell.

[92] Ravandi A., Boekholdt S.M., Mallat Z., Talmud P.J., Kastelein J.J.P., Wareham N.J., Miller E.R., Benessiano J., Tedgui A., Witztum J.L. (2011). Relationship of IgG and IgM Autoantibodies and Immune Complexes to Oxidized LDL with Markers of Oxidation and Inflammation and Cardiovascular Events: Results from the EPIC-Norfolk Study. J. Lipid Res..

[93] Hamze M., Desmetz C., Berthe M.L., Roger P., Boulle N., Brancherau P., Picard E., Guzman C., Tolza C., Guglielmi P. (2013). Characterization of Resident B Cells of Vascular Walls in Human Atherosclerotic Patients. J. Immunol..

[94] Muscari A., Bozzoli C., Gerratana C., Zaca’ F., Rovinetti C., Zauli D., La Placa M., Puddu P. (1988). Association of Serum IgA and C4 with Severe Atherosclerosis. Atherosclerosis.

[95] Major A.S., Fazio S., Linton M.F. (2002). B-Lymphocyte Deficiency Increases Atherosclerosis in LDL Receptor-Null Mice. Arter. Thromb. Vasc. Biol..

[96] Caligiuri G., Nicoletti A., Poirierand B., Hansson G.K. (2002). Protective Immunity against Atherosclerosis Carried by B Cells of Hypercholesterolemic Mice. J. Clin. Investig..

[97] Rosenfeld S.M., Perry H.M., Gonen A., Prohaska T.A., Srikakulapu P., Grewal S., Das D., McSkimming C., Taylor A.M., Tsimikas S. (2015). B-1b Cells Secrete Atheroprotective IgM and Attenuate Atherosclerosis. Circ. Res..

[98] Binder C.J., Hartvigsen K., Chang M.K., Miller M., Broide D., Palinski W., Curtiss L.K., Corr M., Witztum J.L. (2004). IL-5 Links Adaptive and Natural Immunity Specific for Epitopes of Oxidized LDL and Protects from Atherosclerosis. J. Clin. Investig..

[99] RAMSHAW A.L., PARUMS D.V. (1990). Immunohistochemical Characterization of Inflammatory Cells Associated with Advanced Atherosclerosis. Histopathology.

[100] Houtkamp M.A., De Boer O.J., Van Der Loos C.M., Van Der Wal A.C., Becker A.E. (2001). Adventitial Infiltrates Associated with Advanced Atherosclerotic Plaques: Structural Organization Suggests Generation of Local Humoral Immune Responses. J. Pathol..

[101] Engelbertsen D., Rattik S., Wigren M., Vallejo J., Marinkovic G., Schiopu A., Björkbacka H., Nilsson J., Bengtsson E. (2018). IL-1R and MyD88 Signalling in CD4^+^ T Cells Promote Th17 Immunity and Atherosclerosis. Cardiovasc. Res..

[102] Tedgui A., Mallat Z. (2006). Cytokines in Atherosclerosis: Pathogenic and Regulatory Pathways. Physiol. Rev..

[103] Ait-Oufella H., Salomon B.L., Potteaux S., Robertson A.K.L., Gourdy P., Zoll J., Merval R., Esposito B., Cohen J.L., Fisson S. (2006). Natural Regulatory T Cells Control the Development of Atherosclerosis in Mice. Nat. Med..

[104] Adam M., Kooreman N.G., Jagger A., Wagenhäuser M.U., Mehrkens D., Wang Y., Kayama Y., Toyama K., Raaz U., Schellinger I.N. (2018). Systemic Upregulation of IL-10 (Interleukin-10) Using a Nonimmunogenic Vector Reduces Growth and Rate of Dissecting Abdominal Aortic Aneurysm. Arter. Thromb. Vasc. Biol..

[105] Eefting D., Schepers A., De Vries M.R., Pires N.M.M., Grimbergen J.M., Lagerweij T., Nagelkerken L.M., Monraats P.S., Jukema J.W., van Bockel J.H. (2007). The Effect of Interleukin-10 Knock-out and Overexpression on Neointima Formation in Hypercholesterolemic APOE*3-Leiden Mice. Atherosclerosis.

[106] Zhou X., Paulsson G., Stemme S., Hansson G.K. (1998). Hypercholesterolemia Is Associated with a T Helper (Th) 1/Th2 Switch of the Autoimmune Response in Atherosclerotic Apo E-Knockout Mice. J. Clin. Investig..

[107] King V.L., Szilvassy S.J., Daugherty A. (2002). Interleukin-4 Deficiency Decreases Atherosclerotic Lesion Formation in a Site-Specific Manner in Female LDL Receptor-/- Mice. Arter. Thromb. Vasc. Biol..

[108] Davenport P., Tipping P.G. (2003). The Role of Interleukin-4 and Interleukin-12 in the Progression of Atherosclerosis in Apolipoprotein E-Deficient Mice. Am. J. Pathol..

[109] Butcher M.J., Gjurich B.N., Phillips T., Galkina E.V. (2012). The IL-17A/IL-17RA Axis Plays a Proatherogenic Role via the Regulation of Aortic Myeloid Cell Recruitment. Circ. Res..

[110] Smith E., Prasad K.M.R., Butcher M., Dobrian A., Kolls J.K., Ley K., Galkina E. (2010). Blockade of Interleukin-17A Results in Reduced Atherosclerosis in Apolipoprotein E-Deficient Mice. Circulation.

[111] Erbel C., Chen L., Bea F., Wangler S., Celik S., Lasitschka F., Wang Y., Böckler D., Katus H.A., Dengler T.J. (2009). Inhibition of IL-17A Attenuates Atherosclerotic Lesion Development in ApoE-Deficient Mice. J. Immunol..

[112] Danzaki K., Matsui Y., Ikesue M., Ohta D., Ito K., Kanayama M., Kurotaki D., Morimoto J., Iwakura Y., Yagita H. (2012). Interleukin-17A Deficiency Accelerates Unstable Atherosclerotic Plaque Formation in Apolipoprotein e-Deficient Mice. Arter. Thromb. Vasc. Biol..

[113] Santos-Zas I., Lemarié J., Tedgui A., Ait-Oufella H. (2019). Adaptive Immune Responses Contribute to Post-Ischemic Cardiac Remodeling. Front. Cardiovasc. Med..

[114] Nevers T., Salvador A.M., Grodecki-Pena A., Knapp A., Velázquez F., Aronovitz M., Kapur N.K., Karas R.H., Blanton R.M., Alcaide P. (2015). Left Ventricular T-Cell Recruitment Contributes to the Pathogenesis of Heart Failure. Circ. Heart Fail..

[115] Kumar V., Prabhu S.D., Bansal S.S. (2022). CD4^+^ T-Lymphocytes Exhibit Biphasic Kinetics Post-Myocardial Infarction. Front. Cardiovasc. Med..

[116] Kumar V., Rosenzweig R., Asalla S., Nehra S., Prabhu S.D., Bansal S.S. (2022). TNFR1 Contributes to Activation-Induced Cell Death of Pathological CD4^+^ T Lymphocytes During Ischemic Heart Failure. JACC Basic Transl. Sci..

[117] Kyaw T., Winship A., Tay C., Kanellakis P., Hosseini H., Cao A., Li P., Tipping P., Bobik A., Toh B.H. (2013). Cytotoxic and Proinflammatory CD8^+^ T Lymphocytes Promote Development of Vulnerable Atherosclerotic Plaques in ApoE-Deficient Mice. Circulation.

[118] Gotsman I., Grabie N., Dacosta R., Sukhova G., Sharpe A., Lichtman A.H. (2007). Proatherogenic Immune Responses Are Regulated by the PD-1/PD-L Pathway in Mice. J. Clin. Investig..

[119] Gegunde S., Alfonso A., Alvariño R., Alonso E., González-Juanatey C., Botana L.M. (2021). Crosstalk between Cyclophilins and T Lymphocytes in Coronary Artery Disease. Exp. Cell Res..

[120] Netea M.G., Domínguez-Andrés J., Barreiro L.B., Chavakis T., Divangahi M., Fuchs E., Joosten L.A.B., van der Meer J.W.M., Mhlanga M.M., Mulder W.J.M. (2020). Defining Trained Immunity and Its Role in Health and Disease. Nat. Rev. Immunol..

[121] Moore K.J., Sheedy F.J., Fisher E.A. (2013). Macrophages in Atherosclerosis: A Dynamic Balance. Nat. Rev. Immunol..

[122] van der Meer J.W.M., Joosten L.A.B., Riksen N., Netea M.G. (2015). Trained Immunity: A Smart Way to Enhance Innate Immune Defence. Mol. Immunol..

[123] Crișan T.O., Cleophas M.C.P., Oosting M., Lemmers H., Toenhake-Dijkstra H., Netea M.G., Jansen T.L., Joosten L.A.B. (2016). Soluble Uric Acid Primes TLR-Induced Proinflammatory Cytokine Production by Human Primary Cells via Inhibition of IL-1Ra. Ann. Rheum. Dis..

[124] Bekkering S., Quintin J., Joosten L.A.B., van der Meer J.W.M., Netea M.G., Riksen N.P. (2014). Oxidized Low-Density Lipoprotein Induces Long-Term Proinflammatory Cytokine Production and Foam Cell Formation via Epigenetic Reprogramming of Monocytes. Arter. Thromb. Vasc. Biol..

[125] Bekkering S., Arts R.J.W., Novakovic B., Kourtzelis I., van der Heijden C.D.C.C., Li Y., Popa C.D., ter Horst R., van Tuijl J., Netea-Maier R.T. (2018). Metabolic Induction of Trained Immunity through the Mevalonate Pathway. Cell.

[126] Joosten L.A.B., Crişan T.O., Bjornstad P., Johnson R.J. (2020). Asymptomatic Hyperuricaemia: A Silent Activator of the Innate Immune System. Nat. Rev. Rheumatol..

[127] van der Heijden C.D.C.C., Keating S.T., Groh L., Joosten L.A.B., Netea M.G., Riksen N.P. (2019). Aldosterone Induces Trained Immunity: The Role of Fatty Acid Synthesis. Cardiovasc. Res..

[128] Kleinnijenhuis J., Quintin J., Preijers F., Joosten L.A.B., Ifrim D.C., Saeed S., Jacobs C., van Loenhout J., de Jong D., Stunnenberg H.G. (2012). Bacille Calmette-Guérin Induces NOD2-Dependent Nonspecific Protection from Reinfection via Epigenetic Reprogramming of Monocytes. Proc. Natl. Acad. Sci. USA.

[129] Marakalala M.J., Williams D.L., Hoving J.C., Engstad R., Netea M.G., Brown G.D. (2013). Dectin-1 Plays a Redundant Role in the Immunomodulatory Activities of β-Glucan-Rich Ligands in Vivo. Microbes Infect..

[130] Di Luzio N.R., Williams D.L. (1978). Protective Effect of Glucan against Systemic Staphylococcus Aureus Septicemia in Normal and Leukemic Mice. Infect. Immun..

[131] Garly M.-L., Martins C.L., Balé C., Baldé M.A., Hedegaard K.L., Gustafson P., Lisse I.M., Whittle H.C., Aaby P. (2003). BCG Scar and Positive Tuberculin Reaction Associated with Reduced Child Mortality in West Africa. Vaccine.

[132] Braza M.S., van Leent M.M.T., Lameijer M., Sanchez-Gaytan B.L., Arts R.J.W., Pérez-Medina C., Conde P., Garcia M.R., Gonzalez-Perez M., Brahmachary M. (2018). Inhibiting Inflammation with Myeloid Cell-Specific Nanobiologics Promotes Organ Transplant Acceptance. Immunity.

[133] Wendeln A.-C., Degenhardt K., Kaurani L., Gertig M., Ulas T., Jain G., Wagner J., Häsler L.M., Wild K., Skodras A. (2018). Innate Immune Memory in the Brain Shapes Neurological Disease Hallmarks. Nature.

[134] Bekkering S., Joosten L.A.B., van der Meer J.W.M., Netea M.G., Riksen N.P. (2013). Trained Innate Immunity and Atherosclerosis. Curr. Opin. Lipidol..

[135] Arts R.J.W., Joosten L.A.B., Netea M.G. (2018). The Potential Role of Trained Immunity in Autoimmune and Autoinflammatory Disorders. Front. Immunol..

[136] Grigoriou M., Banos A., Filia A., Pavlidis P., Giannouli S., Karali V., Nikolopoulos D., Pieta A., Bertsias G., Verginis P. (2020). Transcriptome Reprogramming and Myeloid Skewing in Haematopoietic Stem and Progenitor Cells in Systemic Lupus Erythematosus. Ann. Rheum. Dis..

[137] Leentjens J., Bekkering S., Joosten L.A.B., Netea M.G., Burgner D.P., Riksen N.P. (2018). Trained Innate Immunity as a Novel Mechanism Linking Infection and the Development of Atherosclerosis. Circ. Res..

[138] Thiem K., Keating S.T., Netea M.G., Riksen N.P., Tack C.J., van Diepen J., Stienstra R. (2021). Hyperglycemic Memory of Innate Immune Cells Promotes In Vitro Proinflammatory Responses of Human Monocytes and Murine Macrophages. J. Immunol..

[139] Edgar L., Akbar N., Braithwaite A.T., Krausgruber T., Gallart-Ayala H., Bailey J., Corbin A.L., Khoyratty T.E., Chai J.T., Alkhalil M. (2021). Hyperglycemia Induces Trained Immunity in Macrophages and Their Precursors and Promotes Atherosclerosis. Circulation.

[140] van der Heijden C.D.C.C., Groh L., Keating S.T., Kaffa C., Noz M.P., Kersten S., van Herwaarden A.E., Hoischen A., Joosten L.A.B., Timmers H.J.L.M. (2020). Catecholamines Induce Trained Immunity in Monocytes In Vitro and In Vivo. Circ. Res..

[141] van Tuijl J., Joosten L.A.B., Netea M.G., Bekkering S., Riksen N.P. (2019). Immunometabolism Orchestrates Training of Innate Immunity in Atherosclerosis. Cardiovasc. Res..

[142] Boffa M.B., Koschinsky M.L. (2019). Oxidized Phospholipids as a Unifying Theory for Lipoprotein(a) and Cardiovascular Disease. Nat. Rev. Cardiol..

[143] Nardin M., Verdoia M., Laera N., Cao D., De Luca G. (2023). New Insights into Pathophysiology and New Risk Factors for ACS. J. Clin. Med..

[144] van der Valk F.M., Bekkering S., Kroon J., Yeang C., Van den Bossche J., van Buul J.D., Ravandi A., Nederveen A.J., Verberne H.J., Scipione C. (2016). Oxidized Phospholipids on Lipoprotein(a) Elicit Arterial Wall Inflammation and an Inflammatory Monocyte Response in Humans. Circulation.

[145] Christ A., Günther P., Lauterbach M.A.R., Duewell P., Biswas D., Pelka K., Scholz C.J., Oosting M., Haendler K., Baßler K. (2018). Western Diet Triggers NLRP3-Dependent Innate Immune Reprogramming. Cell.

[146] Noz M.P., ter Telgte A., Wiegertjes K., Joosten L.A.B., Netea M.G., de Leeuw F.-E., Riksen N.P. (2018). Trained Immunity Characteristics Are Associated with Progressive Cerebral Small Vessel Disease. Stroke.

[147] Bekkering S., van den Munckhof I., Nielen T., Lamfers E., Dinarello C., Rutten J., de Graaf J., Joosten L.A.B., Netea M.G., Gomes M.E.R. (2016). Innate Immune Cell Activation and Epigenetic Remodeling in Symptomatic and Asymptomatic Atherosclerosis in Humans In Vivo. Atherosclerosis.

[148] Kouzarides T. (2007). Chromatin Modifications and Their Function. Cell.

[149] Bannister A.J., Kouzarides T. (2011). Regulation of Chromatin by Histone Modifications. Cell Res..

[150] Saeed S., Quintin J., Kerstens H.H.D., Rao N.A., Aghajanirefah A., Matarese F., Cheng S.-C., Ratter J., Berentsen K., van der Ent M.A. (2014). Epigenetic Programming of Monocyte-to-Macrophage Differentiation and Trained Innate Immunity. Science.

[151] Riksen N.P., Bekkering S., Mulder W.J.M., Netea M.G. (2023). Trained Immunity in Atherosclerotic Cardiovascular Disease. Nat. Rev. Cardiol..

[152] Bekkering S., Stiekema L.C.A., Bernelot Moens S., Verweij S.L., Novakovic B., Prange K., Versloot M., van Lennep J.E.R., Stunnenberg H., de Winther M. (2019). Treatment with Statins Does Not Revert Trained Immunity in Patients with Familial Hypercholesterolemia. Cell Metab..

[153] Keating S.T., Groh L., van der Heijden C.D.C.C., Rodriguez H., dos Santos J.C., Fanucchi S., Okabe J., Kaipananickal H., van Puffelen J.H., Helder L. (2020). The Set7 Lysine Methyltransferase Regulates Plasticity in Oxidative Phosphorylation Necessary for Trained Immunity Induced by β-Glucan. Cell Rep..

[154] Arts R.J.W., Novakovic B., ter Horst R., Carvalho A., Bekkering S., Lachmandas E., Rodrigues F., Silvestre R., Cheng S.-C., Wang S.-Y. (2016). Glutaminolysis and Fumarate Accumulation Integrate Immunometabolic and Epigenetic Programs in Trained Immunity. Cell Metab..

[155] Tercan H., Riksen N.P., Joosten L.A.B., Netea M.G., Bekkering S. (2020). Trained Immunity. Arter. Thromb. Vasc. Biol..

[156] Keating S.T., Groh L., Thiem K., Bekkering S., Li Y., Matzaraki V., van der Heijden C.D.C.C., van Puffelen J.H., Lachmandas E., Jansen T. (2020). Rewiring of Glucose Metabolism Defines Trained Immunity Induced by Oxidized Low-Density Lipoprotein. J. Mol. Med..

[157] Domínguez-Andrés J., Novakovic B., Li Y., Scicluna B.P., Gresnigt M.S., Arts R.J.W., Oosting M., Moorlag S.J.C.F.M., Groh L.A., Zwaag J. (2019). The Itaconate Pathway Is a Central Regulatory Node Linking Innate Immune Tolerance and Trained Immunity. Cell Metab..

[158] Haley M.J., Brough D., Quintin J., Allan S.M. (2019). Microglial Priming as Trained Immunity in the Brain. Neuroscience.

[159] Naik S., Larsen S.B., Gomez N.C., Alaverdyan K., Sendoel A., Yuan S., Polak L., Kulukian A., Chai S., Fuchs E. (2017). Inflammatory Memory Sensitizes Skin Epithelial Stem Cells to Tissue Damage. Nature.

[160] Schnack L., Sohrabi Y., Lagache S.M.M., Kahles F., Bruemmer D., Waltenberger J., Findeisen H.M. (2019). Mechanisms of Trained Innate Immunity in OxLDL Primed Human Coronary Smooth Muscle Cells. Front. Immunol..

[161] Mai J., Virtue A., Shen J., Wang H., Yang X.-F. (2013). An Evolving New Paradigm: Endothelial Cells—Conditional Innate Immune Cells. J. Hematol. Oncol..

[162] El-Osta A., Brasacchio D., Yao D., Pocai A., Jones P.L., Roeder R.G., Cooper M.E., Brownlee M. (2008). Transient High Glucose Causes Persistent Epigenetic Changes and Altered Gene Expression during Subsequent Normoglycemia. J. Exp. Med..

[163] Kiyan Y., Tkachuk S., Hilfiker-Kleiner D., Haller H., Fuhrman B., Dumler I. (2014). OxLDL Induces Inflammatory Responses in Vascular Smooth Muscle Cells via Urokinase Receptor Association with CD36 and TLR4. J. Mol. Cell Cardiol..

[164] Sohrabi Y., Lagache S.M.M., Schnack L., Godfrey R., Kahles F., Bruemmer D., Waltenberger J., Findeisen H.M. (2019). MTOR-Dependent Oxidative Stress Regulates OxLDL-Induced Trained Innate Immunity in Human Monocytes. Front. Immunol..

[165] Cai D., Gao W., Li Z., Zhang Y., Xiao L., Xiao Y. (2022). Current Development of Nano-Drug Delivery to Target Macrophages. Biomedicines.

[166] Mulder W.J.M., Ochando J., Joosten L.A.B., Fayad Z.A., Netea M.G. (2019). Therapeutic Targeting of Trained Immunity. Nat. Rev. Drug Discov..

[167] Shao Y., Cheng Z., Li X., Chernaya V., Wang H., Yang X.F. (2014). Immunosuppressive/Anti-Inflammatory Cytokines Directly and Indirectly Inhibit Endothelial Dysfunction—A Novel Mechanism for Maintaining Vascular Function. J. Hematol. Oncol..

[168] Davidson S.M. (2010). Endothelial Mitochondria and Heart Disease. Cardiovasc. Res..

[169] Garshick M.S., Barrett T.J., Wechter T., Azarchi S., Scher J.U., Neimann A., Katz S., Fuentes-Duculan J., Cannizzaro M.V., Jelic S. (2019). Inflammasome Signaling and Impaired Vascular Health in Psoriasis. Arter. Thromb. Vasc. Biol..

[170] Li Y., Wang D.W., Chen Y., Chen C., Guo J., Zhang S., Sun Z., Ding H., Yao Y., Zhou L. (2018). Genome-Wide Association and Functional Studies Identify SCML4 and THSD7A as Novel Susceptibility Genes for Coronary Artery Disease. Arter. Thromb. Vasc. Biol..

[171] Fetterman J.L., Weisbrod R.M., Feng B., Bastin R., Tuttle S.T., Holbrook M., Baker G., Robertson R.M., Conklin D.J., Bhatnagar A. (2018). Flavorings in Tobacco Products Induce Endothelial Cell Dysfunction. Arter. Thromb. Vasc. Biol..

[172] Sikura K.É., Potor L., Szerafin T., Zarjou A., Agarwal A., Arosio P., Poli M., Hendrik Z., Méhes G., Oros M. (2019). Potential Role of H-Ferritin in Mitigating Valvular Mineralization. Arter. Thromb. Vasc. Biol..

[173] Folco E.J., Mawson T.L., Vromman A., Bernardes-Souza B., Franck G., Persson O., Nakamura M., Newton G., Luscinskas F.W., Libby P. (2018). Neutrophil Extracellular Traps Induce Endothelial Cell Activation and Tissue Factor Production through Interleukin-1α and Cathepsin G. Arter. Thromb. Vasc. Biol..

[174] Segers V.F.M., Brutsaert D.L., De Keulenaer G.W. (2018). Cardiac Remodeling: Endothelial Cells Have More to Say than Just NO. Front. Physiol..

[175] Zhuang T., Liu J., Chen X., Pi J., Kuang Y., Wang Y., Tomlinson B., Chan P., Zhang Q., Li Y. (2019). Cell-Specific Effects of GATA (GATA Zinc Finger Transcription Factor Family)-6 in Vascular Smooth Muscle and Endothelial Cells on Vascular Injury Neointimal Formation. Arter. Thromb. Vasc. Biol..

[176] Fu H., Vadalia N., Xue E.R., Johnson C., Wang L., Yang W.Y., Sanchez C., Nelson J., Chen Q., Choi E.T. (2017). Thrombus Leukocytes Exhibit More Endothelial Cell-Specific Angiogenic Markers than Peripheral Blood Leukocytes Do in Acute Coronary Syndrome Patients, Suggesting a Possibility of Trans-Differentiation: A Comprehensive Database Mining Study. J. Hematol. Oncol..

[177] Lai L., Reineke E., Hamilton D.J., Cooke J.P. (2019). Glycolytic Switch Is Required for Transdifferentiation to Endothelial Lineage. Circulation.

[178] Souilhol C., Harmsen M.C., Evans P.C., Krenning G. (2018). Endothelial–Mesenchymal Transition in Atherosclerosis. Cardiovasc. Res..

[179] Haynes B.A., Yang L.F., Huyck R.W., Lehrer E.J., Turner J.M., Barabutis N., Correll V.L., Mathiesen A., McPheat W., Semmes O.J. (2019). Endothelial-to-Mesenchymal Transition in Human Adipose Tissue Vasculature Alters the Particulate Secretome and Induces Endothelial Dysfunction. Arter. Thromb. Vasc. Biol..

[180] Zhong C., Yang X., Feng Y., Yu J. (2020). Trained Immunity: An Underlying Driver of Inflammatory Atherosclerosis. Front. Immunol..

[181] Wang X., Li Y.-F., Nanayakkara G., Shao Y., Liang B., Cole L., Yang W.Y., Li X., Cueto R., Yu J. (2016). Lysophospholipid Receptors, as Novel Conditional Danger Receptors and Homeostatic Receptors Modulate Inflammation—Novel Paradigm and Therapeutic Potential. J. Cardiovasc. Transl. Res..

[182] Yin Y., Li X., Sha X., Xi H., Li Y.-F., Shao Y., Mai J., Virtue A., Lopez-Pastrana J., Meng S. (2015). Early Hyperlipidemia Promotes Endothelial Activation via a Caspase-1-Sirtuin 1 Pathway. Arter. Thromb. Vasc. Biol..

[183] Mai J., Nanayakkara G., Lopez-Pastrana J., Li X., Li Y.-F., Wang X., Song A., Virtue A., Shao Y., Shan H. (2016). Interleukin-17A Promotes Aortic Endothelial Cell Activation via Transcriptionally and Post-Translationally Activating P38 Mitogen-Activated Protein Kinase (MAPK) Pathway. J. Biol. Chem..

[184] El Kebir D., József L., Pan W., Wang L., Filep J.G. (2009). Bacterial DNA Activates Endothelial Cells and Promotes Neutrophil Adherence through TLR9 Signaling. J. Immunol..

[185] Carman C.V., Martinelli R. (2015). T Lymphocyte–Endothelial Interactions: Emerging Understanding of Trafficking and Antigen-Specific Immunity. Front. Immunol..

[186] Krupnick A.S., Gelman A.E., Barchet W., Richardson S., Kreisel F.H., Turka L.A., Colonna M., Patterson G.A., Kreisel D. (2005). Cutting Edge: Murine Vascular Endothelium Activates and Induces the Generation of Allogeneic CD4^+^25^+^Foxp3^+^ Regulatory T Cells. J. Immunol..

[187] Bedke T., Pretsch L., Karakhanova S., Enk A.H., Mahnke K. (2010). Endothelial Cells Augment the Suppressive Function of CD4^+^CD25^+^Foxp3^+^ Regulatory T Cells: Involvement of Programmed Death-1 and IL-10. J. Immunol..

[188] Gomez D., Owens G.K. (2012). Smooth Muscle Cell Phenotypic Switching in Atherosclerosis. Cardiovasc. Res..

[189] Glagov S., Weisenberg E., Zarins C.K., Stankunavicius R., Kolettis G.J. (1987). Compensatory Enlargement of Human Atherosclerotic Coronary Arteries. N. Engl. J. Med..

[190] Allahverdian S., Chaabane C., Boukais K., Francis G.A., Bochaton-Piallat M.-L. (2018). Smooth Muscle Cell Fate and Plasticity in Atherosclerosis. Cardiovasc. Res..

[191] Schneller M. (1997). Alpha Vbeta 3 Integrin Associates with Activated Insulin and PDGFbeta Receptors and Potentiates the Biological Activity of PDGF. EMBO J..

[192] Englesbe M.J., Deou J., Bourns B.D., Clowes A.W., Daum G. (2004). Interleukin-1β Inhibits PDGF-BB–induced Migration by Cooperating with PDGF-BB to Induce Cyclooxygenase-2 Expression in Baboon Aortic Smooth Muscle Cells. J. Vasc. Surg..

[193] Bhaskar V., Yin J., Mirza A.M., Phan D., Vanegas S., Issafras H., Michelson K., Hunter J.J., Kantak S.S. (2011). Monoclonal Antibodies Targeting IL-1 Beta Reduce Biomarkers of Atherosclerosis in Vitro and Inhibit Atherosclerotic Plaque Formation in Apolipoprotein E-Deficient Mice. Atherosclerosis.

[194] Doran A.C., Yurdagul A., Tabas I. (2020). Efferocytosis in Health and Disease. Nat. Rev. Immunol..

[195] Yurdagul A., Orr A.W. (2016). Blood Brothers: Hemodynamics and Cell–Matrix Interactions in Endothelial Function. Antioxid. Redox Signal.

[196] Feinberg M.W., Watanabe M., Lebedeva M.A., Depina A.S., Hanai J., Mammoto T., Frederick J.P., Wang X.-F., Sukhatme V.P., Jain M.K. (2004). Transforming Growth Factor-Β1 Inhibition of Vascular Smooth Muscle Cell Activation Is Mediated via Smad3. J. Biol. Chem..

[197] Mazighi M., Pellé A., Gonzalez W., Mtairag E.M., Philippe M., Hénin D., Michel J.-B., Feldman L.J. (2004). IL-10 Inhibits Vascular Smooth Muscle Cell Activation in Vitro and in Vivo. Am. J. Physiol-Heart Circ. Physiol..

[198] Kasikara C., Doran A.C., Cai B., Tabas I. (2018). The Role of Non-Resolving Inflammation in Atherosclerosis. J. Clin. Investig..

[199] Lin C.-S., Hsieh P.-S., Hwang L.-L., Lee Y.-H., Tsai S.-H., Tu Y.-C., Hung Y.-W., Liu C.-C., Chuang Y.-P., Liao M.-T. (2018). The CCL5/CCR5 Axis Promotes Vascular Smooth Muscle Cell Proliferation and Atherogenic Phenotype Switching. Cell. Physiol. Biochem..

[200] Chatterjee A., Sharma A., Chen M., Toy R., Mottola G., Conte M.S. (2014). The Pro-Resolving Lipid Mediator Maresin 1 (MaR1) Attenuates Inflammatory Signaling Pathways in Vascular Smooth Muscle and Endothelial Cells. PLoS ONE.

[201] Orr A.W., Lee M.Y., Lemmon J.A., Yurdagul A., Gomez M.F., Schoppee Bortz P.D., Wamhoff B.R. (2009). Molecular Mechanisms of Collagen Isotype-Specific Modulation of Smooth Muscle Cell Phenotype. Arter. Thromb. Vasc. Biol..

[202] Davies M.J., Richardson P.D., Woolf N., Katz D.R., Mann J. (1993). Risk of Thrombosis in Human Atherosclerotic Plaques: Role of Extracellular Lipid, Macrophage, and Smooth Muscle Cell Content. Heart.

[203] Zheng D., Huo M., Li B., Wang W., Piao H., Wang Y., Zhu Z., Li D., Wang T., Liu K. (2021). The Role of Exosomes and Exosomal MicroRNA in Cardiovascular Disease. Front. Cell Dev. Biol..

[204] Baruah J., Wary K.K. (2020). Exosomes in the Regulation of Vascular Endothelial Cell Regeneration. Front. Cell Dev. Biol..

[205] Chen Y.T., Yuan H.X., Ou Z.J., Ou J.S. (2020). Microparticles (Exosomes) and Atherosclerosis. Curr. Atheroscler. Rep..

[206] Barile L., Moccetti T., Marbán E., Vassalli G. (2017). Roles of Exosomes in Cardioprotection. Eur. Heart J..

[207] Guo D., Guo D., Xu Y., Ding J., Dong J., Jia N., Li Y., Zhang M. (2020). Roles and Clinical Applications of Exosomes in Cardiovascular Disease. Biomed. Res. Int..

[208] Fu L., Wu S.S. (2021). Advances in Studies on Exosomes and Microvesicles as Markers of Cardiovascular Disease. Eur. Rev. Med. Pharmacol. Sci..

[209] Sahoo S., Losordo D.W. (2014). Exosomes and Cardiac Repair after Myocardial Infarction. Circ. Res..

[210] Zhan R., Leng X., Liu X., Wang X., Gong J., Yan L., Wang L., Wang Y., Wang X., Qian L.J. (2009). Heat Shock Protein 70 Is Secreted from Endothelial Cells by a Non-Classical Pathway Involving Exosomes. Biochem. Biophys. Res. Commun..

[211] Xie F., Zhan R., Yan L.C., Gong J.B., Zhao Y., Ma J., Qian L.J. (2016). Diet-Induced Elevation of Circulating HSP70 May Trigger Cell Adhesion and Promote the Development of Atherosclerosis in Rats. Cell Stress Chaperones.

[212] Bäck M., Yurdagul A., Tabas I., Öörni K., Kovanen P.T. (2019). Inflammation and Its Resolution in Atherosclerosis: Mediators and Therapeutic Opportunities. Nat. Rev. Cardiol..

[213] Heo J., Kang H. (2022). Exosome-Based Treatment for Atherosclerosis. Int. J. Mol. Sci..

[214] Li S.P., Lin Z.X., Jiang X.Y., Yu X.Y. (2018). Exosomal Cargo-Loading and Synthetic Exosome-Mimics as Potential Therapeutic Tools. Acta Pharmacol. Sin..

[215] Hally K.E., Parker O.M., Brunton-O’Sullivan M.M., Harding S.A., Larsen P.D. (2021). Linking Neutrophil Extracellular Traps and Platelet Activation: A Composite Biomarker Score for Predicting Outcomes after Acute Myocardial Infarction. Thromb. Haemost..

[216] Takahashi M. (2022). NLRP3 Inflammasome as a Key Driver of Vascular Disease. Cardiovasc. Res..

[217] Zeng W., Wu D., Sun Y., Suo Y., Yu Q., Zeng M., Gao Q., Yu B., Jiang X., Wang Y. (2021). The Selective NLRP3 Inhibitor MCC950 Hinders Atherosclerosis Development by Attenuating Inflammation and Pyroptosis in Macrophages. Sci. Rep..

[218] Menu P., Pellegrin M., Aubert J.F., Bouzourene K., Tardivel A., Mazzolai L., Tschopp J. (2011). Atherosclerosis in ApoE-Deficient Mice Progresses Independently of the NLRP3 Inflammasome. Cell Death Dis..

[219] Chen S., Markman J.L., Shimada K., Crother T.R., Lane M., Abolhesn A., Shah P.K., Arditi M. (2020). Sex-Specific Effects of the Nlrp3 Inflammasome on Atherogenesis in LDL Receptor-Deficient Mice. JACC Basic Transl. Sci..

[220] Monticone S., D’Ascenzo F., Moretti C., Williams T.A., Veglio F., Gaita F., Mulatero P. (2018). Cardiovascular Events and Target Organ Damage in Primary Aldosteronism Compared with Essential Hypertension: A Systematic Review and Meta-Analysis. Lancet Diabetes Endocrinol..

[221] Ivanes F., Susen S., Mouquet F., Pigny P., Cuilleret F., Sautière K., Collet J.-P., Beygui F., Hennache B., Ennezat P.V. (2012). Aldosterone, Mortality, and Acute Ischaemic Events in Coronary Artery Disease Patients Outside the Setting of Acute Myocardial Infarction or Heart Failure. Eur. Heart J..

[222] Marzolla V., Armani A., Mammi C., Moss M.E., Pagliarini V., Pontecorvo L., Antelmi A., Fabbri A., Rosano G., Jaffe I.Z. (2017). Essential Role of ICAM-1 in Aldosterone-Induced Atherosclerosis. Int. J. Cardiol..

[223] Caprio M., Newfell B.G., la Sala A., Baur W., Fabbri A., Rosano G., Mendelsohn M.E., Jaffe I.Z. (2008). Functional Mineralocorticoid Receptors in Human Vascular Endothelial Cells Regulate Intercellular Adhesion Molecule-1 Expression and Promote Leukocyte Adhesion. Circ. Res..

[224] Bene N.C., Alcaide P., Wortis H.H., Jaffe I.Z. (2014). Mineralocorticoid Receptors in Immune Cells: Emerging Role in Cardiovascular Disease. Steroids.

[225] Martín-Fernández B., Rubio-Navarro A., Cortegano I., Ballesteros S., Alía M., Cannata-Ortiz P., Olivares-Álvaro E., Egido J., de Andrés B., Gaspar M.L. (2016). Aldosterone Induces Renal Fibrosis and Inflammatory M1-Macrophage Subtype via Mineralocorticoid Receptor in Rats. PLoS ONE.

[226] Usher M.G., Duan S.Z., Ivaschenko C.Y., Frieler R.A., Berger S., Schütz G., Lumeng C.N., Mortensen R.M. (2010). Myeloid Mineralocorticoid Receptor Controls Macrophage Polarization and Cardiovascular Hypertrophy and Remodeling in Mice. J. Clin. Investig..

[227] Rickard A.J., Morgan J., Tesch G., Funder J.W., Fuller P.J., Young M.J. (2009). Deletion of Mineralocorticoid Receptors from Macrophages Protects against Deoxycorticosterone/Salt-Induced Cardiac Fibrosis and Increased Blood Pressure. Hypertension.

[228] Raz-Pasteur A., Gamliel-Lazarovich A., Gantman A., Coleman R., Keidar S. (2014). Mineralocorticoid Receptor Blockade Inhibits Accelerated Atherosclerosis Induced by a Low Sodium Diet in Apolipoprotein E-Deficient Mice. J. Renin Angiotensin Aldosterone Syst..

[229] Shen Z.-X., Chen X.-Q., Sun X.-N., Sun J.-Y., Zhang W.-C., Zheng X.-J., Zhang Y.-Y., Shi H.-J., Zhang J.-W., Li C. (2017). Mineralocorticoid Receptor Deficiency in Macrophages Inhibits Atherosclerosis by Affecting Foam Cell Formation and Efferocytosis. J. Biol. Chem..

[230] Zhu X., Li Q., George V., Spanoudis C., Gilkes C., Shrestha N., Liu B., Kong L., You L., Echeverri C. (2023). A Novel Interleukin-2-Based Fusion Molecule, HCW9302, Differentially Promotes Regulatory T Cell Expansion to Treat Atherosclerosis in Mice. Front. Immunol..

[231] Abbas A.K., Trotta E., Simeonov D.R., Marson A., Bluestone J.A. (2018). Revisiting IL-2: Biology and Therapeutic Prospects. Sci. Immunol..

[232] Kabata H., Moro K., Koyasu S. (2018). The Group 2 Innate Lymphoid Cell (ILC2) Regulatory Network and Its Underlying Mechanisms. Immunol. Rev..

[233] Huang Y., Paul W.E. (2015). Inflammatory Group 2 Innate Lymphoid Cells. Int. Immunol..

[234] Newland S.A., Mohanta S., Clément M., Taleb S., Walker J.A., Nus M., Sage A.P., Yin C., Hu D., Kitt L.L. (2017). Type-2 Innate Lymphoid Cells Control the Development of Atherosclerosis in Mice. Nat. Commun..

[235] Eberl G., Colonna M., Di Santo J.P., McKenzie A.N.J. (2015). Innate Lymphoid Cells: A New Paradigm in Immunology. Science.

[236] Rak G.D., Osborne L.C., Siracusa M.C., Kim B.S., Wang K., Bayat A., Artis D., Volk S.W. (2016). IL-33-Dependent Group 2 Innate Lymphoid Cells Promote Cutaneous Wound Healing. J. Investig. Dermatol..

[237] Brestoff J.R., Kim B.S., Saenz S.A., Stine R.R., Monticelli L.A., Sonnenberg G.F., Thome J.J., Farber D.L., Lutfy K., Seale P. (2015). Group 2 Innate Lymphoid Cells Promote Beiging of White Adipose Tissue and Limit Obesity. Nature.

[238] Zhao T.X., Newland S.A., Mallat Z. (2020). 2019 ATVB Plenary Lecture. Arterioscler. Thromb. Vasc. Biol..

[239] Ahmadzadeh M., Rosenberg S.A. (2006). IL-2 Administration Increases CD4^+^CD25hi Foxp3+ Regulatory T Cells in Cancer Patients. Blood.

[240] Rosenzwajg M., Lorenzon R., Cacoub P., Pham H.P., Pitoiset F., El Soufi K., RIbet C., Bernard C., Aractingi S., Banneville B. (2019). Immunological and Clinical Effects of Low-Dose Interleukin-2 across 11 Autoimmune Diseases in a Single, Open Clinical Trial. Ann. Rheum. Dis..

[241] Zhao T.X., Kostapanos M., Griffiths C., Arbon E.L., Hubsch A., Kaloyirou F., Helmy J., Hoole S.P., Rudd J.H.F., Wood G. (2018). Low-Dose Interleukin-2 in Patients with Stable Ischaemic Heart Disease and Acute Coronary Syndromes (LILACS): Protocol and Study Rationale for a Randomised, Double-Blind, Placebo-Controlled, Phase I/II Clinical Trial. BMJ Open.

[242] Chen L., Flies D.B. (2013). Molecular Mechanisms of T Cell Co-Stimulation and Co-Inhibition. Nat. Rev. Immunol..

[243] Topalian S.L., Taube J.M., Anders R.A., Pardoll D.M. (2016). Mechanism-Driven Biomarkers to Guide Immune Checkpoint Blockade in Cancer Therapy. Nat. Rev. Cancer.

[244] Tan S., Day D., Nicholls S.J., Segelov E. (2022). Immune Checkpoint Inhibitor Therapy in Oncology. JACC CardioOncol.

[245] Ramos-Casals M., Brahmer J.R., Callahan M.K., Flores-Chávez A., Keegan N., Khamashta M.A., Lambotte O., Mariette X., Prat A., Suárez-Almazor M.E. (2020). Immune-Related Adverse Events of Checkpoint Inhibitors. Nat. Rev. Dis. Primers.

[246] Ridker P.M., Everett B.M., Thuren T., MacFadyen J.G., Chang W.H., Ballantyne C., Fonseca F., Nicolau J., Koenig W., Anker S.D. (2017). Antiinflammatory Therapy with Canakinumab for Atherosclerotic Disease. N. Engl. J. Med..

[247] Ewing M.M., Karper J.C., Abdul S., de Jong R.C.M., Peters H.A.B., de Vries M.R., Redeker A., Kuiper J., Toes R.E.M., Arens R. (2013). T-Cell Co-Stimulation by CD28–CD80/86 and Its Negative Regulator CTLA-4 Strongly Influence Accelerated Atherosclerosis Development. Int. J. Cardiol..

[248] Ma K., Lv S., Liu B., Liu Z., Luo Y., Kong W., Xu Q., Feng J., Wang X. (2013). CTLA4-IgG Ameliorates Homocysteine-Accelerated Atherosclerosis by Inhibiting T-Cell Overactivation in ApoE^−/−^ Mice. Cardiovasc. Res..

[249] Poels K., van Leent M.M.T., Reiche M.E., Kusters P.J.H., Huveneers S., de Winther M.P.J., Mulder W.J.M., Lutgens E., Seijkens T.T.P. (2020). Antibody-Mediated Inhibition of CTLA4 Aggravates Atherosclerotic Plaque Inflammation and Progression in Hyperlipidemic Mice. Cells.

[250] Li S.-H., Chen W.-J., Yan M., Shu Y.-W., Liao Y.-H. (2015). Expression of Coinhibitory PD-L1 on CD4^+^CD25^+^FOXP3^+^ Regulatory T Cells Is Elevated in Patients with Acute Coronary Syndrome. Coron. Artery Dis..

[251] Fernandez D.M., Rahman A.H., Fernandez N.F., Chudnovskiy A., Amir E.D., Amadori L., Khan N.S., Wong C.K., Shamailova R., Hill C.A. (2019). Single-Cell Immune Landscape of Human Atherosclerotic Plaques. Nat. Med..

